# Omega-3 Fatty Acids Regulate Mammary Gland Lipogenesis and Development via Gα_s_-Mediated cAMP–EPAC Signaling Pathway

**DOI:** 10.34133/research.0767

**Published:** 2025-07-08

**Authors:** Baofeng Li, Senlin Su, Siyu Yuan, Dongpang Chen, Qianzi Zhang, Qihui Li, Xiaoai Lin, Xiaohuan Liang, Wutai Guan, Shihai Zhang

**Affiliations:** ^1^ Guangdong Province Key Laboratory of Animal Nutrition Control, Guangzhou 510642, China.; ^2^ College of Animal Science, South China Agricultural University, Guangzhou 510642, China.; ^3^ College of Veterinary Medicine, South China Agricultural University, Guangzhou 510642, China.; ^4^ National Engineering Research Center for Breeding Swine Industry, South China Agricultural University, Guangzhou 510642, China.; ^5^ Guangdong Laboratory for Lingnan Modern Agriculture, South China Agricultural University, Guangzhou 510642, China.

## Abstract

G protein-coupled receptor 120 (GPR120) plays a pivotal role in regulating lactation, yet its underlying mechanisms remain unclear. In mouse models, GPR120 expression in the mammary gland increases markedly during lactation. Under inflammatory conditions, both n-3 polyunsaturated fatty acids (n-3 PUFAs) and GPR120 agonists markedly reduced inflammatory responses and enhanced lipogenesis and migration in HC11 mammary epithelial cells. These benefits were also observed under non-inflammatory conditions and were diminished when GPR120 was knocked down. Furthermore, the regulatory function of GPR120 under non-inflammatory conditions in in vivo and in vitro models is explored. We discovered that the GPR120–Gα_s_–cyclic adenosine monophosphate (cAMP)–exchange protein directly activated by cAMP (EPAC) signaling axis is critical for lipogenesis and migration in mammary epithelial cells. Through transcriptomic analyses, the EPAC–CCCTC-binding factor (CTCF)–peroxisome proliferator-activated receptor γ (PPARγ)/CCAAT enhancer-binding protein α (C/EBPα) pathway was identified to primarily govern lipogenesis, while the EPAC–C-X-C motif chemokine ligand 14 (CXCL14)/C-X-C chemokine receptor type 4 (CXCR4) autocrine loop regulates migration of mammary epithelial cells. Overall, these findings suggest that GPR120, which can be activated by n-3 PUFAs, improves mammary gland performance by alleviating inflammation and directly modulating mammary lipogenesis and mammary gland development through the CTCF–PPARγ/C/EBPα and CXCL14–CXCR4 pathways. Thus, GPR120 and its downstream signaling targets may represent an important clinical target for enhancing maternal lactation.

## Introduction

During lactation, the mammary gland undergoes significant structural and functional changes to produce milk, which is essential for neonatal nutrition and early immune development [1–3]. Although maternal intake of n-3 polyunsaturated fatty acids (PUFAs) has been shown to improve mammary gland function, the underlying molecular mechanisms remain unclear [4,5]. Because the lactating mammary gland experiences intense milk synthesis and secretion, as well as oxidative and inflammatory stress [2,6], uncovering how n-3 fatty acids regulate mammary gland function is critical for enhancing milk quality and supporting healthy offspring development.

G protein-coupled receptors (GPCRs) are a central family of transmembrane signaling molecules involved in metabolism, secretion, migration, differentiation, and immune responses [7–9]. G protein-coupled receptor 120 (GPR120) and GPR40, in particular, sense long-chain fatty acids and link dietary lipid intake to metabolic balance by regulating inflammation, insulin secretion, and other metabolic processes [7,10]. While GPR120 has been shown to mitigate inflammation via the GPR120–β-arrestin2 pathway, [11], it is not yet clear whether its role in lactation depends solely on this anti-inflammatory effect or also involves other signaling pathways that govern mammary gland development and mammary lipogenesis [12]. Meanwhile, although GPR40 exhibits important functions in various tissues, its specific role in the mammary gland, particularly in mammary lipogenesis and mammary gland development, remains to be determined.

In this study, we found that both GPR120 and GPR40 participate in regulating mammary gland function in response to n-3 PUFAs, with GPR120 playing a dominant role. Under inflammatory conditions, GPR120 reduces the secretion of proinflammatory mediators, thereby improving mammary gland development and mammary lipogenesis. Additionally, under non-inflammatory conditions, GPR120 regulates mammary gland homeostasis through the GPR120–Gα_s_–cyclic adenosine monophosphate (cAMP)–exchange protein directly activated by cAMP (EPAC) axis. Specifically, the EPAC–CCCTC-binding factor (CTCF)–peroxisome proliferator-activated receptor γ (PPARγ)/CCAAT enhancer-binding protein α (C/EBPα) branch is critical for mammary lipogenesis, while the EPAC–C-X-C motif chemokine ligand 14 (CXCL14)/C-X-C chemokine receptor type 4 (CXCR4) autocrine loop predominantly governs mammary gland development. These findings underscore the importance of GPR120 in n-3 PUFA-mediated regulation of mammary gland migration and lactation, suggesting that GPR120 and its downstream signaling targets hold potential as clinical interventions to enhance maternal lactation capacity.

## Results

### Expression dynamics and anti-inflammatory roles of GPR120 and GPR40 during mammary gland lactation

**Fig. 1.**
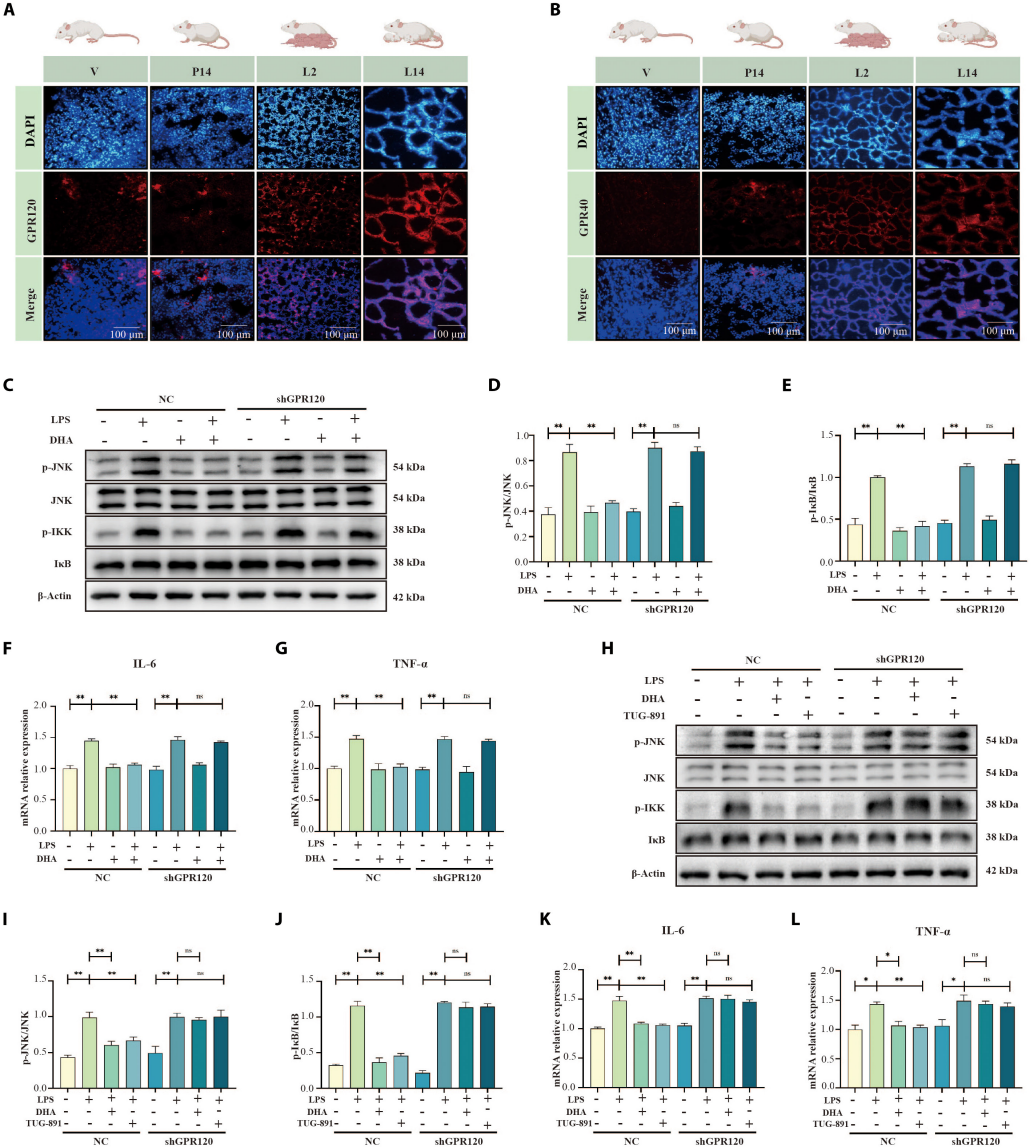
Expression patterns of GPR120 and GPR40 in the mammary gland and the anti-inflammatory role of GPR120. (A and B) Immunofluorescence images showing the expression levels of GPR120 and GPR40 in the mammary gland at different stages: virgin (V), day 14 of pregnancy (P14), day 2 of lactation (L2), and day 14 of lactation (L14). (C to E) Western blot analysis of inflammation-related signaling proteins (JNK/p-JNK and IκB/p-IKK) in HC11 cells with shRNA-mediated GPR120 knockdown, treated with DHA (100 μM) and/or LPS (25 μg/ml), *n* = 3. (F and G) mRNA expression levels of inflammatory cytokines (IL-6 and TNF-α) in HC11 cells with shRNA-GPR120 knockdown, treated with DHA and/or LPS, *n* = 3. (H to J) Western blot analysis of inflammation-related signaling proteins (JNK/p-JNK and IκB/p-IKK) in HC11 cells with shRNA-GPR120 knockdown, treated with DHA, the GPR120 agonist TUG-891(50 μM), and/or LPS, *n* = 3. (K and L) mRNA expression levels of inflammatory cytokines (IL-6 and TNF-α) in HC11 cells with shRNA-GPR120 knockdown, treated with DHA, TUG-891, and/or LPS, *n* = 3. *P < 0.05, **P < 0.01. ns, not significant.

To clarify the roles of GPR120 and GPR40 in mammary gland development, we assessed their expression patterns at various developmental stages using immunofluorescence. Both receptors exhibited steadily increasing expression levels throughout lactation (Fig. 1A and B), indicating potential regulatory functions. We then investigated whether *GPR120* and *GPR40* enhance mammary gland function by mitigating lactation-associated inflammation. In HC11 cells with *GPR120* or *GPR40* knocked down (Fig. S1A to C), the GPR120 agonist TUG-891 showed a marked anti-inflammatory effect that was significantly reduced following *GPR120* knockdown (Fig. S1D to H; *P* < 0.01). By contrast, the GPR40 agonist TAK-875 did not produce a notable anti-inflammatory response (Fig. S1I to M). Under inflammatory conditions, treatment with the natural GPR120 agonist docosahexaenoic acid (DHA; 22: 6n-3) markedly suppressed the inflammatory pathway [evidenced by decreased phosphorylation of c-Jun N-terminal kinase (JNK) and inhibitor of nuclear factor κB (NF-κB) (IκB)] (Fig. 1C to E) and lowered interleukin-6 (IL-6) and tumor necrosis factor α (TNF-α) levels (Fig. 1F and G; *P* < 0.01). The anti-inflammatory effects of both DHA and TUG-891 were lost when *GPR120* was knocked down (Fig. 1H to L).

**Fig. 2.**
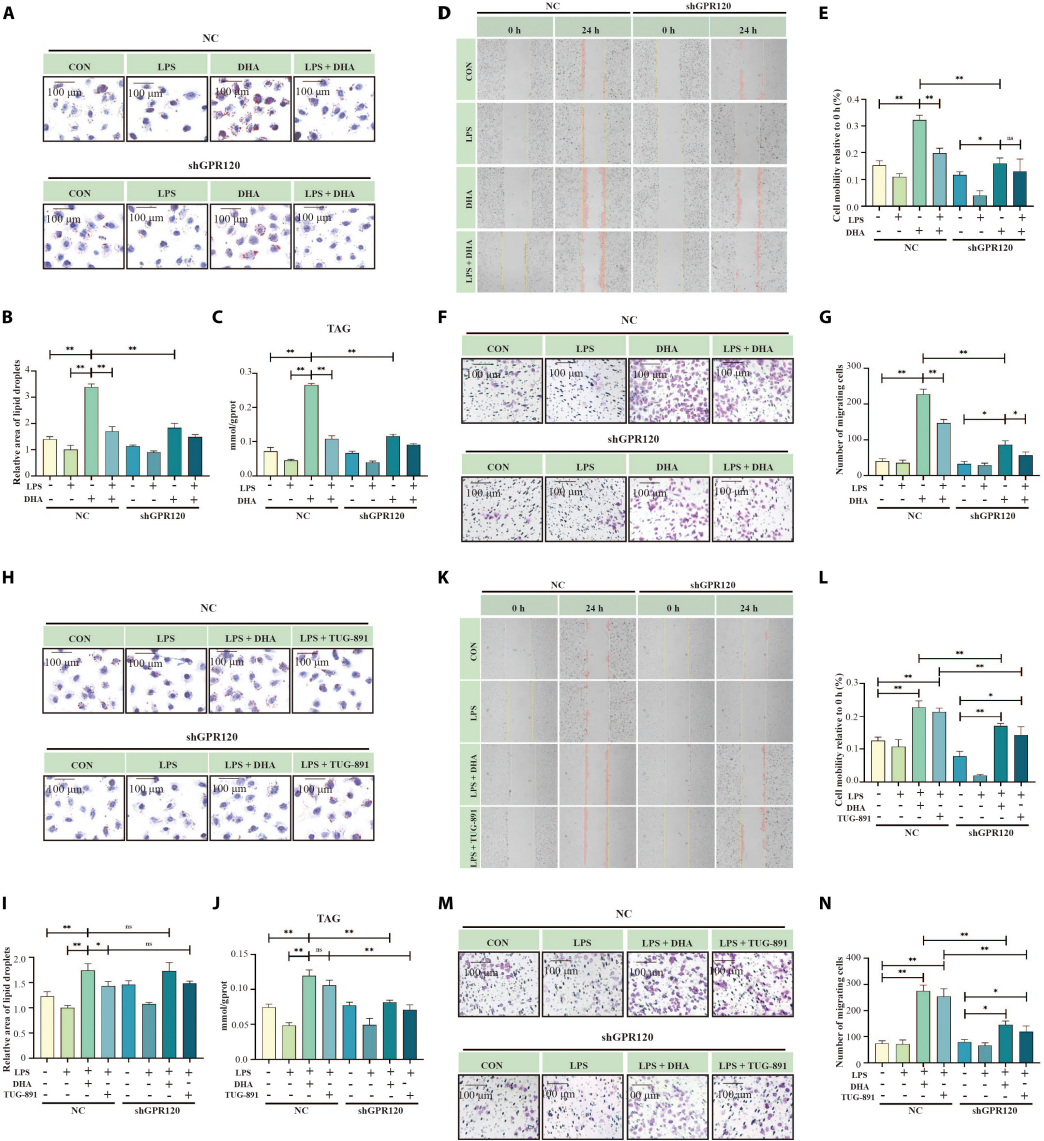
Effects of DHA and GPR120 agonists on mammary lipogenesis and cell migration under inflammatory conditions. (A and B) Oil Red O staining of HC11 cells treated with DHA (100 μM) and/or LPS (25 μg/ml) under shRNA-GPR120 conditions, *n* = 3. Scale bar, 100 μm. (C) Triglyceride quantification in HC11 cells treated with DHA and/or LPS under shRNA-GPR120 conditions, *n* = 3. (D and E) Cell scratch assay results of HC11 cells treated with DHA and/or LPS under shRNA-GPR120 conditions, *n* = 3. (F and G) Transwell assay of HC11 cells treated with DHA and/or LPS under shRNA-GPR120 conditions, *n* = 3. Scale bar, 100 μm. (H and I) Oil Red O staining of HC11 cells treated with DHA, TUG-891 (50 μM), and/or LPS under shRNA-GPR120 conditions, *n* = 3. Scale bar, 100 μm. (J) Triglyceride quantification in HC11 cells treated with DHA, TUG-891, and/or LPS under shRNA-GPR120 conditions, *n* = 3. (K and L) Cell scratch assay results of HC11 cells treated with DHA, TUG-891, and/or LPS under shRNA-GPR120 conditions, *n* = 3. (M and N) Transwell assay of HC11 cells treated with DHA, TUG-891, and/or LPS under shRNA-GPR120 conditions, *n* = 3. Scale bar, 100 μm. *P < 0.05, **P < 0.01.

To further explore how GPR120 and GPR40 influence mammary epithelial function under inflammatory conditions, we examined their effects on lipid synthesis, as well as cell migration and invasion, in HC11 cells. DHA significantly increased lipid droplet formation (Fig. 2A and B), triglyceride levels (Fig. 2C), and cell migration (Fig. 2D to G; *P* < 0.01) under inflammatory conditions. However, these benefits were notably reduced following GPR120 knockdown. Similarly, TUG-891 elevated lipid synthesis (Fig. 2H to J) and cell migration and invasion (Fig. 2K to N) under inflammatory conditions. Intriguingly, even in the absence of inflammation, DHA regulated lipid synthesis and promoted cell migration and invasion (Fig. 2A to G), suggesting that DHA can directly influence mammary gland function through non-inflammatory pathways [13].

### Regulation of mammary gland function by DHA under non-inflammatory conditions

**Fig. 3.**
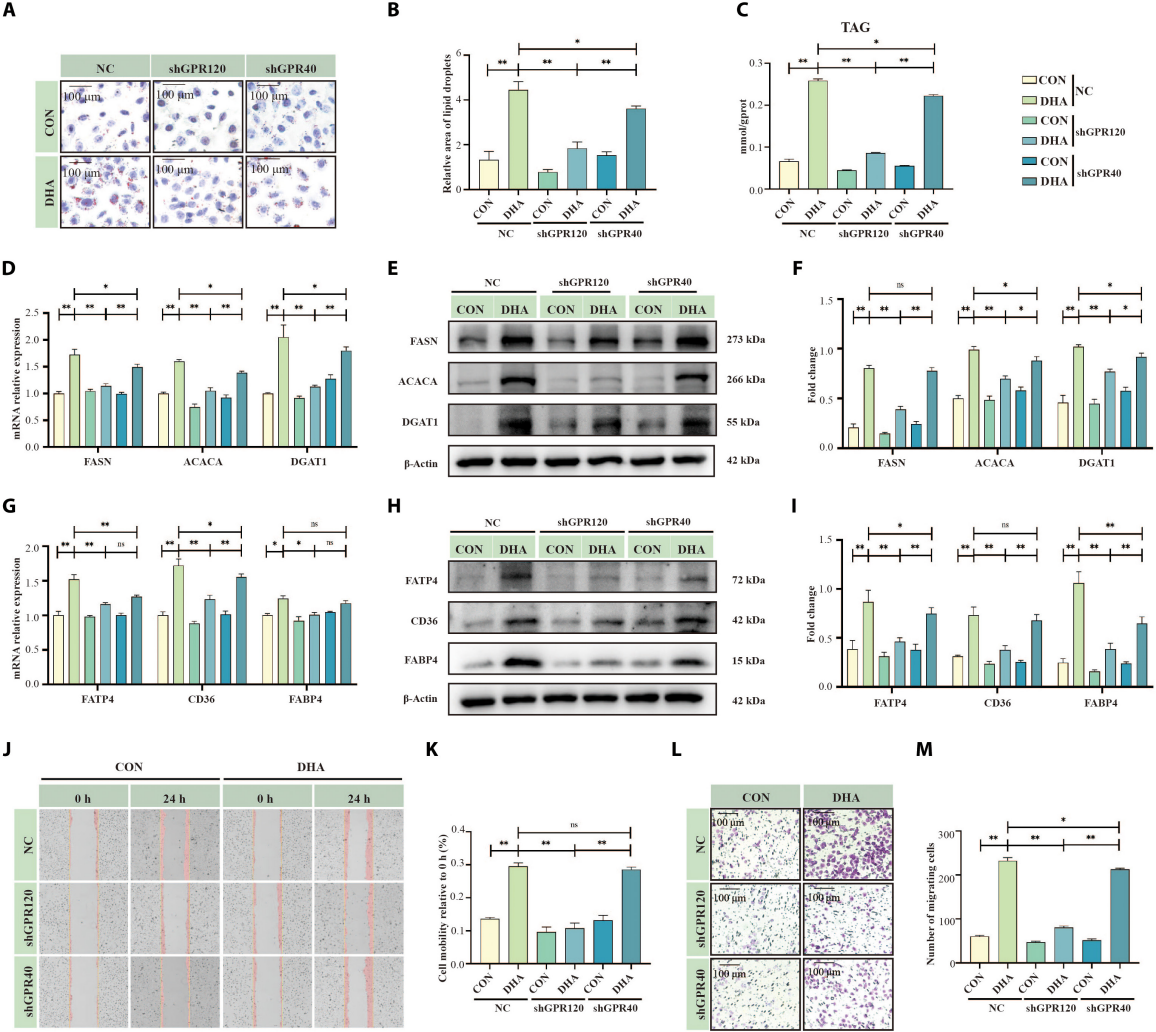
Impact of GPR120/GPR40 knockdown on DHA-induced mammary lipogenesis and cell migration. (A and B) Oil Red O staining of HC11 cells treated with DHA under shRNA-GPR120/40 conditions, *n* = 3. Scale bar, 100 μm. (C) Triglyceride quantification in HC11 cells treated with DHA (100 μM) under shRNA-GPR120/40 conditions, *n* = 3. (D) mRNA expression of lipid synthesis proteins in HC11 cells treated with DHA under shRNA-GPR120/40 conditions, *n* = 3. (E and F) Western blot analysis of lipid synthesis proteins in HC11 cells treated with DHA under shRNA-GPR120/40 conditions, *n* = 3. (G) mRNA expression of lipid transport proteins in HC11 cells treated with DHA under shRNA-GPR120/40 conditions, *n* = 3. (H and I) Western blot analysis of lipid transport proteins in HC11 cells treated with DHA under shRNA-GPR120/40 conditions, *n* = 3. (J and K) Cell scratch assay results of HC11 cells treated with DHA under shRNA-GPR120/40 conditions, *n* = 3. (L and M) Transwell assay of HC11 cells treated with DHA under shRNA-GPR120/40 conditions, *n* = 3. Scale bar, 100 μm. *P < 0.05, **P < 0.01.

To determine whether GPR120 or GPR40 signaling directly mediates mammary gland function, we evaluated their roles under non-inflammatory conditions. Knocking down *GPR120* or *GPR40* in HC11 cells significantly reduced DHA-induced lipid droplet formation (Fig. 3A and B; *P* < 0.05) and triglyceride levels (Fig. 3C; *P* < 0.05). Consistently, genes and proteins involved in lipid synthesis [fatty acid synthase (FASN), acetyl-CoA carboxylα (ACACA), diacylglycerol O-acyltransferase 1 (DGAT1); Fig. 3D to F] and lipid transport [fatty acid transporter 4 (FATP4), platelet glycoprotein 4 (CD36), fatty acid binding protein 4 (FABP4); Fig. 3G to I] were markedly down-regulated, with the strongest effects observed following *GPR120* knockdown (*P* < 0.01). Wound healing and Transwell assays demonstrated a pronounced decline in DHA-induced cell migration and invasion when *GPR120* was knocked down (Fig. 3J to M; *P* < 0.01), compared with a moderate decrease after *GPR40* knockdown (Fig. 3L and M; *P* < 0.05). In line with this, the GPR120 inhibitor AH-7614 more effectively suppressed DHA-induced lipid synthesis (Fig. S2A to I) and cell migration and invasion (Fig. S2J to M; *P* < 0.01) than the GPR40 inhibitor GW-1100. Moreover, while both GPR120 (TUG-891) and GPR40 (TAK-875) agonists enhanced milk fat synthesis and cell migration and invasion, TUG-891 produced a stronger effect (Fig. S2N to Z; *P* < 0.05).

To confirm that these outcomes were not merely due to the addition of fatty acids, we compared DHA (an omega-3 PUFA) with palmitic acid (PA; a saturated fatty acid) and linoleic acid (LA; an omega-6 PUFA). DHA consistently produced the most pronounced increases in lipid synthesis, transport, and cell migration and invasion (Fig. S3A to M; *P* < 0.05). Notably, DHA also significantly elevated GPR120 expression relative to the other tested fatty acids (Fig. S3N and O; *P* < 0.05). These results collectively indicate that DHA can directly regulate milk fat synthesis as well as cell migration and invasion through GPR120, independent of inflammatory pathways [4,14]

### Role of the GPR120–Gα_s_–cAMP–EPAC axis in mammary lipogenesis and mammary gland development

**Fig. 4.**
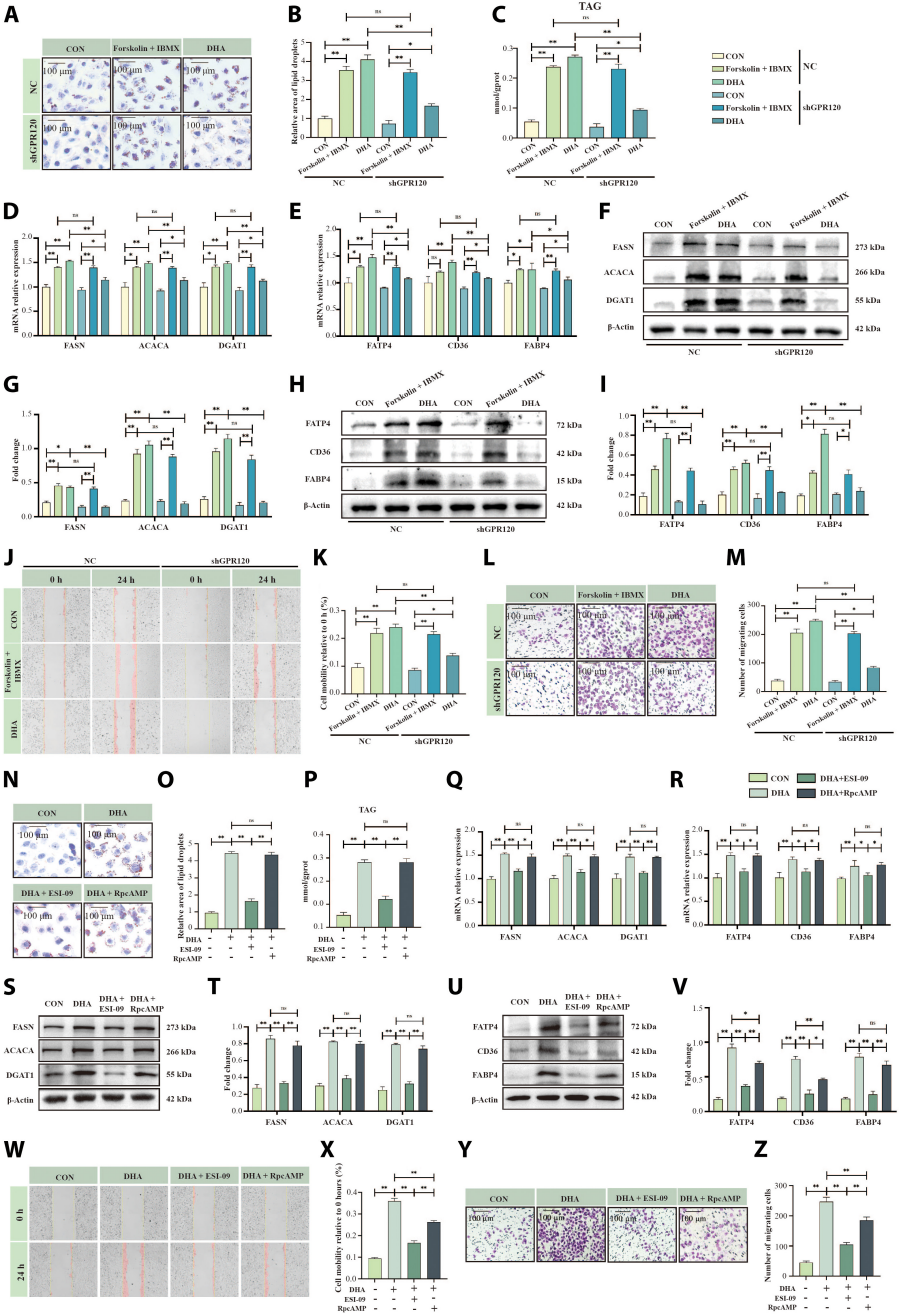
Effects of cAMP and downstream EPAC/PKA signaling on milk fat synthesis and cell migration. (A and B) Oil Red O staining of HC11 cells treated with DHA (100 μM), forskolin (20 μM), and IBMX (20 μM) (cAMP mimetics) under shRNA-GPR120 conditions, *n* = 3. Scale bar, 100 μm. (C) Triglyceride quantification in HC11 cells treated with DHA, forskolin, and IBMX under shRNA-GPR120 conditions, *n* = 3. (D and E) mRNA expression levels of lipid synthesis and transport proteins in HC11 cells treated with DHA, forskolin, and IBMX under shRNA-GPR120 conditions, *n* = 3. (F to I) Western blot analysis of lipid synthesis and transport proteins in HC11 cells treated with DHA, forskolin, and IBMX under shRNA-GPR120 conditions, *n* = 3.(J and K) Cell scratch assay results of HC11 cells treated with DHA, forskolin, and IBMX under shRNA-GPR120 conditions, *n* = 3. (L and M) Transwell assay of HC11 cells treated with DHA, forskolin, and IBMX under shRNA-GPR120 conditions, *n* = 3. Scale bar, 100 μm. (N to Q) Oil Red O staining of HC11 cells treated with DHA, ESI-09 (EPAC inhibitor, 20 μM), and RpcAMP (PKA inhibitor, 20 μM), *n* = 3. Scale bar, 100 μm. (P) Triglyceride quantification in HC11 cells treated with DHA, ESI-09, and RpcAMP, *n* = 3. (Q and R) mRNA expression levels of lipid synthesis and transport proteins in HC11 cells treated with DHA, ESI-09, and RpcAMP, *n* = 3. (S to V) Western blot analysis of lipid synthesis and transport proteins in HC11 cells treated with DHA, ESI-09, and RpcAMP, *n* = 3. (W and X) Cell scratch assay results of HC11 cells treated with DHA, ESI-09, and RpcAMP, *n* = 3. (Y and Z) Transwell assay of HC11 cells treated with DHA, ESI-09, and RpcAMP, *n* = 3. Scale bar, 100 μm. *P < 0.05, **P < 0.01.

We next investigated how GPR120 activates downstream signaling. Unlike GPR40, GPR120 interacts with Gα_s_, leading to elevated cAMP and subsequent activation of key pathways. Forskolin (an adenylyl cyclase activator) and IBMX (a phosphodiesterase inhibitor), both of which increase intracellular cAMP [11], served as positive controls to mimic GPR120–Gα_s_ activation. We observed that DHA and forskolin + IBMX promoted lipid synthesis in HC11 cells; however, the effect of DHA was significantly reduced when *GPR120* was knocked down. In contrast, forskolin + IBMX partially rescued lipid synthesis under *GPR120* knockdown, confirming that DHA relies on GPR120-mediated cAMP signaling to enhance lipid production (Fig. 4A to C; *P* < 0.01). Consistent with these results, *GPR120* knockdown significantly reduced the expression of genes and proteins involved in DHA-induced lipid synthesis (FASN, ACACA, DGAT1) and transport (FATP4, CD36, FABP4), but forskolin + IBMX restored these levels (Fig. 4D to I; *P* < 0.01). Moreover, wound healing and Transwell assays demonstrated that DHA promotes cell migration and invasion by elevating cAMP through GPR120 in HC11 cells (Fig. 4J to M; *P* < 0.01).

EPAC and protein kinase A (PKA) represent 2 major downstream pathways of cAMP [15]. To further validate the GPR120–Gα_s_–cAMP axis, we selectively inhibited EPAC (ESI-09) or PKA (RpcAMP) [15]. Notably, EPAC inhibition led to substantially greater reductions in DHA-induced lipid droplet formation (Fig. 4N to P), lipid synthesis and transport gene/protein expression (Fig. 4Q to V), and cell migration and invasion (Fig. 4W to Z; *P* < 0.01) compared with PKA inhibition. Dose–response assays (Fig. S4A to F) reinforced these observations, highlighting EPAC as the key downstream mediator of GPR120–Gα_s_–cAMP signaling.

### Transcriptomic screening of EPAC downstream target genes

**Fig. 5.**
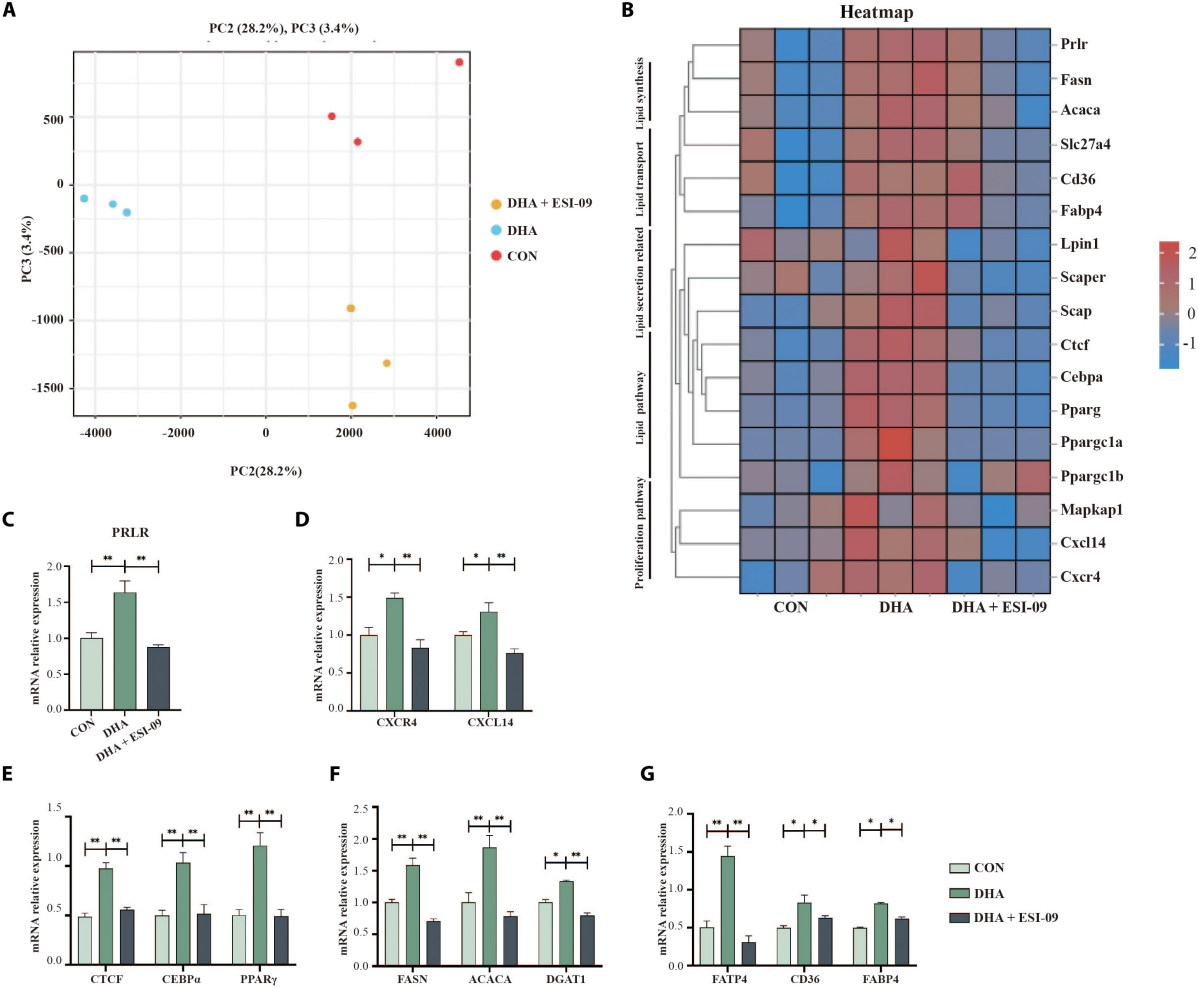
Transcriptome analysis and qPCR validation in HC11 cells treated with DHA and EPAC inhibitor ESI-09. (A) Principal components analysis (PCA) of the transcriptome, *n* = 3. HC11 cells were treated with DHA and ESI-09 (EPAC inhibitor, 20 μM). (B) Heatmap of transcriptome analysis, *n* = 3. HC11 cells were treated with DHA and ESI-09. (C) mRNA expression of PRLR in HC11 cells treated with DHA and ESI-09, *n* = 3. (D) mRNA expression of migration signaling proteins in HC11 cells treated with DHA and ESI-09, *n* = 3. (E) mRNA expression of lipid pathway proteins in HC11 cells treated with DHA and ESI-09, *n* = 3. (F and G) mRNA expression of lipid synthesis and transport proteins in HC11 cells treated with DHA and ESI-09, *n* = 3. *P < 0.05, **P < 0.01.

To identify the EPAC-regulated factors involved in milk fat synthesis and cell migration and invasion, we conducted transcriptomic analyses. Principal components analysis showed distinct gene expression profiles among the CON, DHA, and DHA + ESI-09 (EPAC inhibitor) groups (Fig. 5A). Further differential expression analysis showed that DHA treatment significantly up-regulated genes associated with lipid metabolism, including *FASN*, *ACACA*, *FATP4*, *CD36*, *FABP4*, *CTCF*, *PPARγ*, and *C/EBPα*, as well as genes related to cell migration and invasion, such as *CXCL14* and *CXCR4*. Notably, cotreatment with ESI-09 reversed these DHA-induced transcriptional changes, supporting a regulatory role for EPAC in mediating both lipid metabolism and cellular motility pathways (Fig. 5B).

Among these genes, *prolactin receptor* (*PrlR*) emerged as a key candidate, reflecting its crucial role in prolactin sensitivity. Follow-up experiments confirmed that DHA increases PrlR expression via the GPR120–cAMP–EPAC pathway (Fig. 5C and Fig. S4G to J). We further validated changes in genes linked to cell migration and invasion (Fig. 5D) and fat synthesis (Fig. 5E to G) through reverse transcription polymerase chain reaction (RT-PCR) (*P* < 0.05). Collectively, these findings highlight the central role of EPAC in regulating mammary epithelial cell function and identify PrlR, along with genes involved in cell migration, invasion, and fat synthesis, as critical downstream targets.

### The critical role of the EPAC–CTCF–PPARγ/C/EBPα axis in mammary lipogenesis

**Fig. 6.**
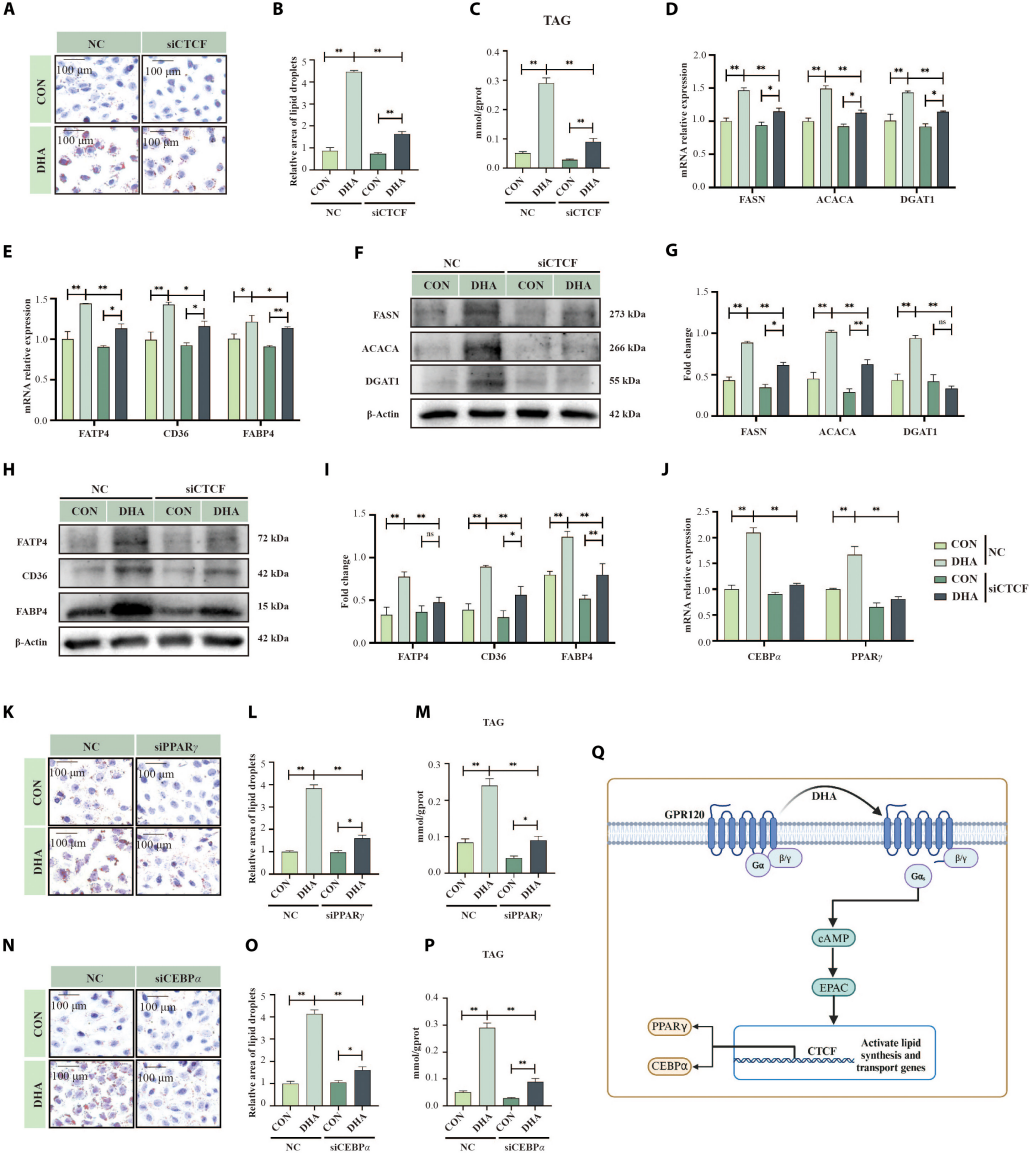
EPAC–CTCF–PPARγ/C/EBPα pathway regulates mammary lipogenesis. (A and B) Oil Red O staining of HC11 cells treated with DHA (100 μM) under siRNA-CTCF knockdown conditions, *n* = 3. Scale bar, 100 μm. (C) Triglyceride quantification in HC11 cells treated with DHA under siRNA-CTCF knockdown conditions, *n* = 3. (D and E) Western blot analysis of lipid synthesis and transport proteins in HC11 cells treated with DHA under siRNA-CTCF knockdown conditions, *n* = 3. (F to I) Western blot analysis of lipid synthesis and transport proteins in HC11 cells treated with DHA under siRNA-CTCF knockdown conditions, *n* = 3. (J) mRNA expression of downstream factors C/EBPα and PPARγ in HC11 cells following CTCF knockdown and DHA treatment, *n* = 3. (K and L) Oil Red O staining of HC11 cells treated with DHA under PPARγ knockdown conditions, *n* = 3. Scale bar, 100 μm. (M) Triglyceride quantification in HC11 cells treated with DHA under PPARγ knockdown conditions, *n* = 3. (N and O) Oil Red O staining of HC11 cells treated with DHA under C/EBPα knockdown conditions, *n* = 3. Scale bar, 100 μm. (P) Triglyceride quantification in HC11 cells treated with DHA under C/EBPα knockdown conditions, *n* = 3. (Q) Proposed pathway of the GPR120–Gα_s_–cAMP–EPAC–CTCF–PPARγ/C/EBPα axis in milk fat synthesis. *P < 0.05, **P < 0.01.

To investigate the role of CTCF, a known upstream regulator of *PPARγ* and *C/EBPα*, in DHA-induced lipogenic regulation, we performed targeted knockdown of *CTCF*, *PPARγ*, and *C/EBPα* in HC11 mammary epithelial cells and evaluated their effects on milk fat synthesis. Because *CTCF* can influence chromatin conformation and thereby regulate other functional genes [16], we first silenced CTCF (Fig. S5A and B). This intervention significantly reduced DHA-induced milk fat synthesis, as indicated by lower lipid droplet accumulation and triglyceride levels (Fig. 6A to C; *P* < 0.01) in HC11 cells. Genes involved in lipid synthesis (*FASN*, *ACACA*, *DGAT1*) and transport (*FATP4*, *CD36*, *FABP4*) were also markedly down-regulated (Fig. 6D and E; *P* < 0.05), and their protein levels followed a similar pattern (Fig. 6F to I; *P* < 0.01). Further analysis revealed that CTCF silencing weakened DHA-induced up-regulation of *PPARγ* and *C/EBPα* (Fig. 6J; *P* < 0.01) [17], both of which play important roles in lipid synthesis. Notably, knocking down *PPARγ* or *C/EBPα* (Fig. S5C to F) also impaired DHA-induced milk fat synthesis in HC11 cells (Fig. 6K to P; *P* < 0.01). Taken together, these findings underscore the significance of the CTCF–PPARγ/C/EBPα axis in DHA-mediated milk fat synthesis and demonstrate that DHA can directly regulate this process under non-inflammatory conditions through the EPAC–CTCF–PPARγ/C/EBPα pathway (Fig. 6Q).

### The EPAC–CXCL14/CXCR4 autocrine pathway and mammary epithelial migration and invasion

**Fig. 7.**
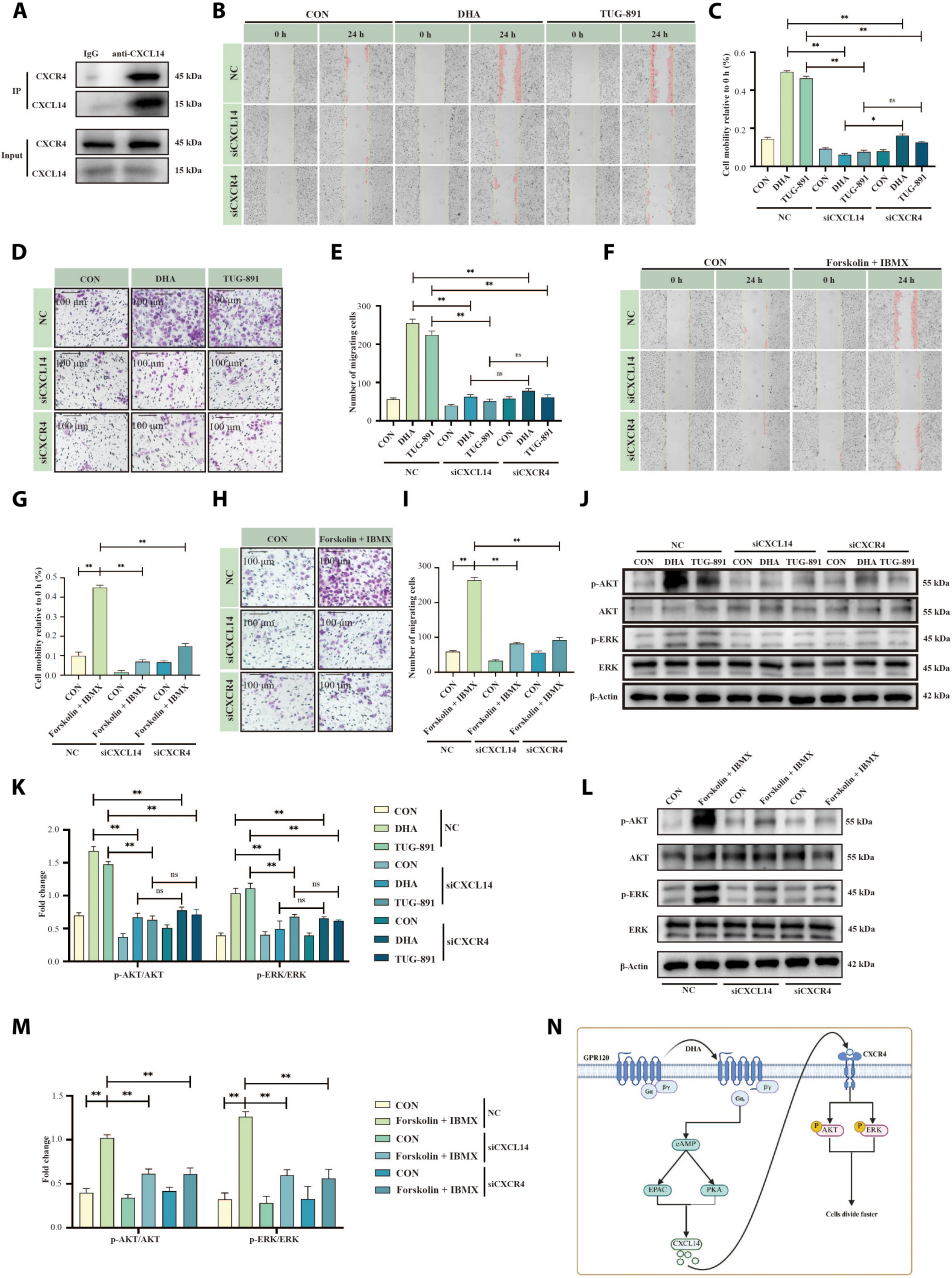
cAMP-CXCL14/CXCR4 pathway regulates mammary epithelial cell migration. (A) Coimmunoprecipitation Western blot analysis of CXCL14 and CXCR4 interaction. (B and C) Cell scratch assay of HC11 cells treated with DHA (100 μM) or TUG-891 (GPR120 agonist, 50 μM)) under siRNA-CXCL14 or siRNA-CXCR4 knockdown conditions, *n* = 3. (D and E) Transwell migration assay of HC11 cells treated with DHA or TUG-891 under siRNA-CXCL14 or siRNA-CXCR4 knockdown conditions, *n* = 3. Scale bar, 100 μm. (F and G) Cell scratch assay of HC11 cells treated with forskolin and IBMX (cAMP mimetics) under siRNA-CXCL14 or siRNA-CXCR4 knockdown conditions, *n* = 3. (H and I) Transwell migration assay of HC11 cells treated with forskolin and IBMX under siRNA-CXCL14 or siRNA-CXCR4 knockdown conditions, *n* = 3. Scale bar, 100 μm. (J and K) Western blot analysis of migration-related proteins in HC11 cells treated with DHA or TUG-891 under siRNA-CXCL14 or siRNA-CXCR4 knockdown conditions, *n* = 3. (L and M) Western blot analysis of migration-related proteins in HC11 cells treated with forskolin and IBMX under siRNA-CXCL14 or siRNA-CXCR4 knockdown conditions, *n* = 3. (N) Proposed pathway illustrating the GPR120–Gα_s_–cAMP–EPAC/PKA–CXCL14–CXCR4–AKT/ERK axis in milk fat synthesis. *P < 0.05, **P < 0.01.

We next examined whether DHA regulates mammary epithelial cell migration and invasion through the CXCL14/CXCR4 pathway [18,19]. Coimmunoprecipitation confirmed a direct interaction between CXCL14 and CXCR4 (Fig. 7A). Wound healing and Transwell assays showed that knocking down either *CXCL14* or *CXCR4* (Fig. S5G to J) significantly weakened the cell migration and invasion induced by DHA and the GPR120 agonist TUG-891 (Fig. 7B to E; *P* < 0.01). Similarly, when *CXCL14* or *CXCR4* was knocked down, cAMP-induced cell migration and invasion were also diminished (Fig. 7F to I; *P* < 0.01), underscoring the pivotal role of CXCL14/CXCR4 in these processes. In addition to CXCR4, atypical chemokine receptor 2 (ACKR2) and GPR85 have been identified as potential CXCL14 receptors [20,21]. To exclude their involvement, we knocked them down in HC11 cells and found that silencing *ACKR2* or *GPR85* had a comparatively minor impact on DHA-induced cell migration and invasion (Fig. S5K to O; *P* < 0.01). Moreover, *CXCL14* depletion markedly reduced *CXCR4* expression while exerting only minor effects on *ACKR2* and *GPR85* levels (Fig. S5P; *P* < 0.01). These findings underscore CXCL14/CXCR4 as the primary pathway driving cell migration and invasion.

AKT and extracellular signal-regulated kinase (ERK) are key downstream signaling molecules of CXCR4 that play major roles in cell migration and invasion [22]. Knockdown of *CXCL14* or *CXCR4* significantly decreased DHA-induced protein kinase B (AKT) and ERK phosphorylation (Fig. 7J to M; *P* < 0.01). In addition, specific inhibitors of AKT and ERK markedly suppressed DHA-induced cell migration and invasion (Fig. S5Q to T; *P* < 0.01). Collectively, these results demonstrate that DHA-mediated mammary epithelial cell migration and invasion are governed by the cAMP–EPAC–CXCL14/CXCR4–AKT/ERK axis (Fig. 7N).

### Maternal DHA supplementation regulates mammary gland development and mammary lipogenesis through a GPR120-dependent signaling pathway

**Fig. 8.**
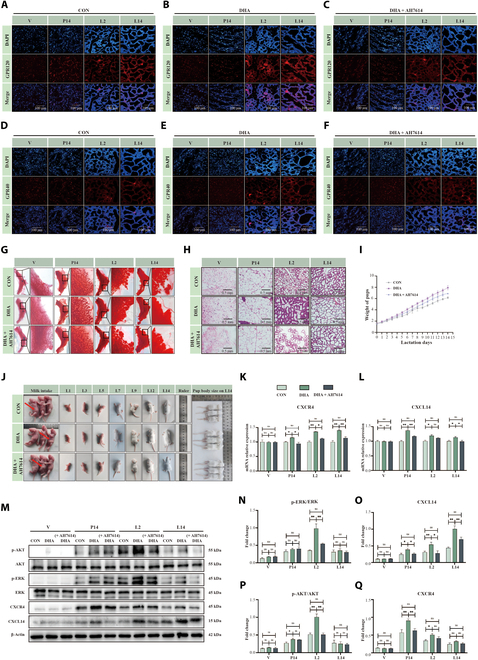
Maternal DHA supplementation modulates mammary gland development and offspring growth through the GPR120-CXCL14/CXCR4 pathway. (A to F) Expression patterns of GPR120 and GPR40 in mammary glands at various physiological stages: virgin (V), day 14 of pregnancy (P14), day 2 of lactation (L2), and day 14 of lactation (L14). Mice were treated with DHA (200 mg every 2 d, oral gavage) and AH-7614 (GPR120 inhibitor, 50 μg every 2 d, intraperitoneal injection). (G) Whole-mount analysis of mammary glands across different physiological stages. (H) H&E staining of mammary alveoli at different physiological stages, *n* = 3. Scale bar, 0.5 mm. (I and J) Growth performance metrics of offspring pups. (K and L) mRNA expression levels of migration-related genes in mammary glands at different physiological stages (V, P14, L2, and L14), *n* = 3. (M to Q) Western blot analysis of migration-related proteins in mammary glands at various physiological stages (V, P14, L2, and L14), *n* = 3. *P < 0.05, **P < 0.01.

In mice, DHA supplementation during lactation increased the expression of GPR120 and GPR40 in the mammary gland, with GPR120 up-regulated more markedly than GPR40 (Fig. 8A to F), and promoted mammary cell migration, invasion, and differentiation (Fig. 8G), as well as enhanced alveolar structure formation (Fig. 8H). These effects were diminished when GPR120 was inhibited. Correspondingly, offspring from DHA-fed dams grew more rapidly and reached larger sizes (Fig. 8I and J). Consistently, DHA supplementation elevated CXCL14 and CXCR4 protein levels (Fig. 8K and L; *P* < 0.05) and increased AKT and ERK phosphorylation (Fig. 8M to Q; *P* < 0.05), which were reversed by GPR120 inhibition.

**Fig. 9.**
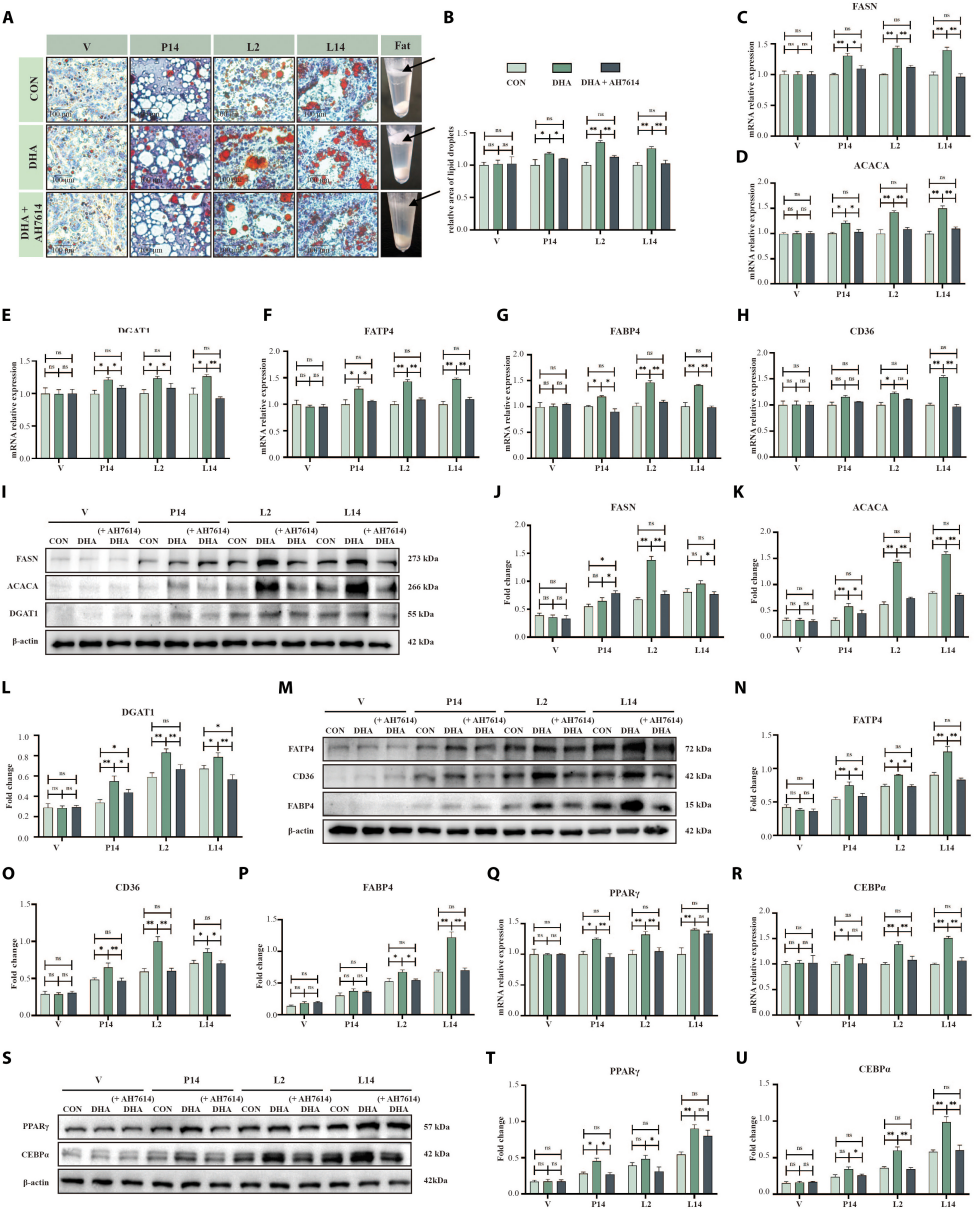
Maternal DHA supplementation modulate mammary lipogenesis through GPR120-PPARγ/C/EBPα pathway in mammary glands. (A and B) Distribution of lipid droplets in mammary glands and milk fat content during lactation at different physiological stages: virgin (V), day 14 of pregnancy (P14), day 2 of lactation (L2), and day 14 of lactation (L14). Mice were treated with DHA (200 mg every 2 d, oral gavage) and AH-7614 (GPR120 inhibitor, 50 μg every 2 d, intraperitoneal injection), *n* = 3. Scale bar, 100 μm. (C to H) mRNA expression levels of lipid synthesis and transport proteins in mammary glands across different physiological stages, *n* = 3. (I to P) Western blot analysis of lipid synthesis and transport proteins in mammary glands across different physiological stages, *n* = 3. (Q and R) mRNA expression levels of lipid signaling pathway proteins (PPARγ and C/EBPα) in mammary glands across different physiological stages, *n* = 3. (S to U) Western blot analysis of lipid signaling pathway proteins (PPARγ and C/EBPα) in mammary glands across different physiological stages, *n* = 3. *P < 0.05, **P < 0.01.

DHA supplementation also led to a marked rise in lipid droplets within mammary alveoli and higher overall milk lipid content (Fig. 9A and B). Consistent with this, genes involved in fatty acid synthesis (*FASN*, *ACACA*, *DGAT1*) and lipid transport (*FATP4*, *CD36*) were up-regulated at gestation day 14 (G14), lactation day 2 (L2), and lactation day 14 (L14) (Fig. 9C to H; *P* < 0.05), with corresponding increases at the protein level (Fig. 9I to P; *P* < 0.05). The transcription factors PPARγ and C/EBPα were similarly elevated (Fig. 9Q to U; *P* < 0.05). Inhibiting GPR120 reversed these effects, underscoring its essential role in DHA–GPR120 signaling in lactation.

## Discussion

Acute and chronic mastitis adversely impact maternal mammary health and can result in abnormal milk composition [23–25]. In our study, using a lipopolysaccharide (LPS)-induced in vitro mastitis model [26], we observed a substantial reduction in lipid synthesis and cell migration in mammary epithelial cells. Previous research has shown that activating GPR120 reduces inflammation in macrophages through the TAK1–β-arrestin2/TAB1 pathway [7,27]. Similarly, we found that n-3 PUFAs inhibit LPS-induced inflammatory signaling and decrease the release of inflammatory factors in mammary epithelial cells by activating GPR120. Additionally, we discovered that GPR120 can directly regulate cell migration and lipid synthesis in mammary epithelial cells even under non-inflammatory conditions.

To elucidate the biological role of GPR120, we measured its expression at various stages of pregnancy: early pregnancy, the onset of lactation, and peak lactation on day 14. GPR120 expression steadily increased throughout pregnancy and lactation. We also examined GPR40, another long-chain fatty acid receptor, and observed a similar expression pattern. However, activation of GPR120 produced a greater enhancement of lipogenesis and cell migration than activation of GPR40 in mammary epithelial cells, suggesting that GPR120 is the primary GPCR regulating lactation. It has been reported that both GPR120 and GPR40 interact with Gα_q/11_ proteins, but only GPR120 specifically binds to Gα_s_ [28,29]. This suggests that the Gα_s_ pathway is essential for GPR120-mediated lipid synthesis.

Prolactin receptor (PRLR) activation primarily regulates mammary development, lipid synthesis, milk protein secretion, and maternal behaviors through the Janus kinase 2 (JAK2)–signal transducer and activator of transcription (STAT5) signaling pathway [30–32]. Our transcriptomic analysis identified PRLR as a key target influenced by the GPR120–Gα_s_–cAMP–EPAC axis. Additionally, we identified the CTCF–PPARγ/C/EBPα pathway as crucial for lipid synthesis. Disruption of this pathway severely impaired GPR120’s ability to regulate lipid synthesis in mammary gland epithelial cells, which is similar to how GPR120 activation regulates lipogenesis in adipocyte precursors [33]. Furthermore, we discovered that the EPAC–CXCL14/CXCR4 autocrine loop plays a role in regulating mammary epithelial cell migration and differentiation. Previous studies have shown that mammary basal cells secrete CXCL14, which binds to receptors on mammary epithelial cells and thereby promotes their migration and differentiation [20,22]. In our study, we found that activation of GPR120 stimulates the secretion of CXCL14 from mammary epithelial cells, which in turn activates CXCR4 within the same cells, promoting their growth and differentiation [34]. This reveals a novel autocrine mechanism by which GPR120 enhances mammary gland function.

Our in vivo mouse experiments confirmed the in vitro findings, demonstrating that n-3 PUFAs promote mammary epithelial cell growth and differentiation through GPR120, thereby improving lactation performance. Studies in other species also support the role of n-3 PUFAs in regulating lactation [35–39]. These results suggest that incorporating fish oil into the diet could be an effective nutritional strategy to enhance maternal lactation and maintain mammary gland health, in both inflammatory and non-inflammatory conditions [35,40].

**Fig. 10.**
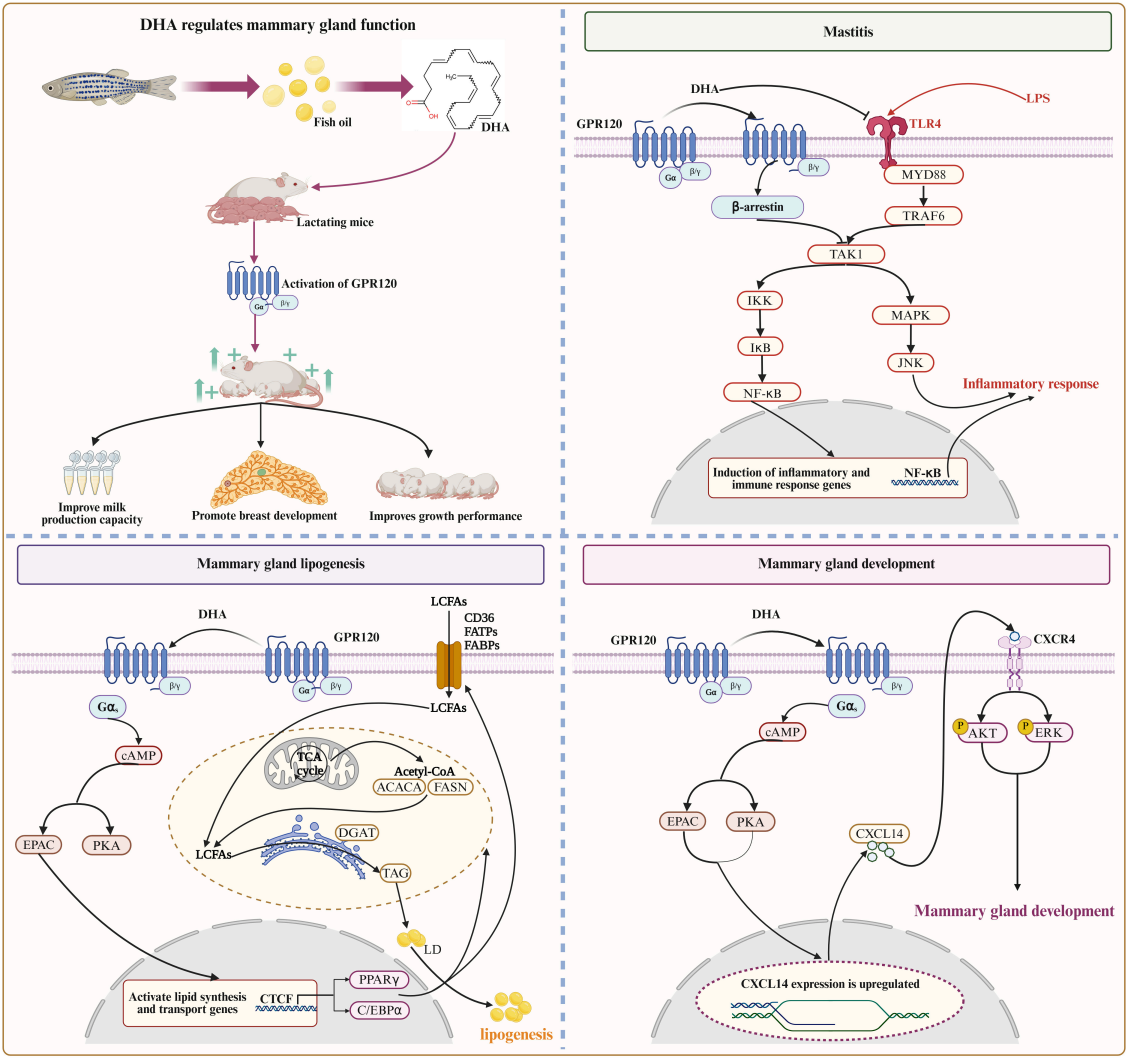
Working model of GPR120 modulation of mammary gland function. GPR120 enhances mammary gland function by modulating inflammation, lipogenesis, and development. GPR120 expression in the mammary gland rises substantially during lactation, suggesting a key role in supporting milk production. GPR120 signals through the Gα_s_–cAMP–EPAC pathway to enhance mammary lipogenesis and mammary gland development. Gene expression studies indicate that the EPAC–CTCF–PPARγ/CEBPα branch primarily regulates mammary lipogenesis, while the EPAC–CXCL14/CXCR4 autocrine loop drives mammary gland development. Overall, GPR120 activation, especially by n-3 PUFAs, improves mammary function by mitigating inflammation and directly controlling mammary lipogenesis and mammary gland development, highlighting a promising clinical target to enhance lactation.

Overall, our study highlights the crucial role of GPR120 in mediating the beneficial effects of n-3 PUFAs on mammary gland function. By elucidating the GPR120–Gα_s_–cAMP–EPAC signaling axis and its downstream pathways, we provide a comprehensive understanding of how dietary fatty acids influence lactation. These insights offer potential clinical targets for improving maternal lactation capacity and ensuring the healthy development of offspring (Fig. 10).

## Materials and Methods

### Animals

A total of 128 female ICR mice (5 weeks old) were obtained from Guangdong SJA Laboratory Animal Technology Co. Ltd. (Guangzhou, China). All animals were housed under controlled environmental conditions, including a constant temperature of 25 °C, relative humidity of 50% to 60%, and a 12-h light/dark cycle (light: 07:30 to 19:30; dark: 19:30 to 07:30). Mice were fed a standard growth and breeding diet [specific pathogen-free (SPF)-grade, irradiation sterilized; product no. 1010083] provided by Jiangsu Synergy Pharmaceutical Bioengineering Co. Ltd. All animal procedures were approved by the Animal Experimentation Center of South China Agricultural University (approval number: 2023c080-1).

### Animal experiment design

Experiment 1: Thirty-two female ICR mice (5 weeks old) were acclimated to the diet and housing conditions for 1 week. At 7 weeks of age, the mice were mated, and the day of successful mating was designated as gestation day 0 (G0). Mammary gland and tissue samples were collected at G14, L2, and L14.

Experiment 2: Ninety-six female ICR mice (5 weeks old) were randomly assigned to 3 groups (*n* = 32 per group) and acclimated under identical conditions for 1 week. Virgin-phase samples were collected at 6 weeks of age. At 7 weeks of age, all mice were bred and simultaneously subjected to treatment interventions. The control group received vehicle treatment; the DHA group was administered 200 mg of docosahexaenoic acid (DHA; Sigma) by oral gavage every 2 d; and the DHA + GPR120 inhibitor group received the same DHA dosage by gavage, along with 50 μg of AH-7614 (a selective GPR120 inhibitor) administered intraperitoneally every 2 d.

### Mammary gland sampling

Mice were euthanized in a standardized supine position at the indicated lactation stage. To ensure anatomical consistency and enable multiple downstream applications from the same animal, the entire fourth and fifth pairs of mammary glands were harvested and processed as follows: The right fourth inguinal mammary gland was fixed in Carnoy’s fixative (ethanol:chloroform:acetic acid, 6:3:1, v/v/v) for whole-mount staining. The left fourth gland was embedded in optimal cutting temperature (OCT) compound (Leica, Germany) and snap-frozen for cryosectioning. Both procedures preserved full gland architecture for morphological and immunofluorescent analyses. The fifth pair of mammary glands was used for molecular and histological analyses. The left fifth mammary gland was fixed in 4% paraformaldehyde (PFA) for 24 h at 4 °C and paraffin-embedded for histological evaluation. The right fifth gland was snap-frozen in liquid nitrogen and stored at –80 °C for RNA and protein extraction used in quantitative PCR (qPCR) and Western blot assays.

### Breast tissue immunofluorescence

Paraffin-embedded breast tissues were sectioned (5 μm thickness), deparaffinized with xylene, and rehydrated through a series of graded ethanol solutions. Antigen retrieval was performed using 50× EDTA antigen retrieval solution (pH 8.0) at sub-boiling temperature for 10 min. After blocking with 3% bovine serum albumin in phosphate-buffered saline for 30 min, the sections were incubated overnight at 4 °C with primary antibodies. Fluorescent secondary antibodies were applied for 30 min, followed by nuclear counterstaining with 4′,6-diamidino-2-phenylindole (DAPI) (Sigma, USA). Slides were observed under a confocal fluorescence microscope (Nikon, Japan). The antibodies used for both Western blot and immunofluorescence assays are summarized in Table S1.

### Whole-mount staining of breast tissue

Fresh, intact breast tissue was placed on glass slides and fixed in Carnoy’s reagent for 12 h. After fixation, the tissue was transferred to acetone and soaked for 2 d to remove fat and other extraneous tissues. The whole mount was sequentially soaked in 100%, 95%, and 70% ethanol, followed by distilled water for 60 min. The tissue was stained in carmine solution for 4 h and then rinsed in a 2% hydrochloric acid–70% ethanol solution for 1 d. The samples were rehydrated through ethanol solutions and immersed in xylene overnight, after which they were observed and photographed.

### Hematoxylin and eosin staining of breast tissue

Breast tissue samples were fixed, dehydrated through a series of graded ethanol solutions, embedded in paraffin, and cut into 5-μm sections. The sections were stained with hematoxylin and eosin (H&E) (Saville, China) and then dehydrated and sealed.

### Mammary Oil Red O staining

Tissue embedded in OCT (Sakura, USA) was sectioned (10 μm) using a cryomicrotome, and sections were fixed with 4% PFA for 30 min. Oil Red O staining was performed for 15 min, followed by rinsing with 60% isopropanol solution to remove excess stain. The nuclei were counterstained with hematoxylin, and the samples were sealed with glycerin gelatin (Saville, China) before being observed under a microscope.

### Milk collection

Milk was collected on the 14th day of lactation. Prior to collection, pups were separated from the mother. The mice were anesthetized with tribromoethanol, and 500 μl of milk was collected using a 1-ml syringe. The milk was then centrifuged at 3,000 rpm for 20 min, and the fat layer was separated.

### HC11 cell culture, differentiation, and treatment

HC11 mouse mammary epithelial cells were cultured in complete growth medium consisting of Dulbecco’s modified Eagle’s medium (DMEM)/F12 supplemented with 10 ng/ml epidermal growth factor (EGF), 5 μg/ml insulin-like growth factor 1 (IGF-1), 5 μg/ml insulin–transferrin–selenium (ITS), 10% fetal bovine serum (FBS), and 1% penicillin–streptomycin (PS). Cells were maintained at 37 °C in a humidified atmosphere containing 5% CO₂. To induce lactogenic differentiation, cells were seeded and allowed to reach 70% to 80% confluence. Differentiation was initiated by replacing the growth medium with EGF-free medium containing 10% FBS for 24 h, followed by incubation in hormone-stimulated differentiation medium composed of DMEM/F12 supplemented with 1 μM dexamethasone, 5 μg/ml insulin, 5 μg/ml prolactin, and 10% FBS for the indicated durations.

For experiments assessing mammary lipogenesis, small interfering RNA (siRNA)/short hairpin RNA (shRNA) transfections and pharmacological treatments were performed at the beginning of the differentiation phase, immediately following EGF withdrawal. For cell migration and invasion assays, undifferentiated HC11 cells maintained under standard growth conditions were used for siRNA/shRNA transfections and pharmacological treatments. The sources and catalog numbers of all treatment reagents are listed in Table S2.

### Establishing inflammatory cell models using LPS

To investigate inflammatory responses in proliferative mammary epithelial cells, undifferentiated HC11 cells were cultured in complete medium and allowed to reach approximately 90% confluence. Cells were then treated with 5 μg/ml LPS in complete medium for 4 h.

To assess the impact of inflammation on mammary lipogenesis, differentiated HC11 cells were exposed to LPS during the lactogenic phase. After 24 h of EGF withdrawal, cells were cultured in hormone-stimulated differentiation medium containing 1 μM dexamethasone, 5 μg/ml insulin, 5 μg/ml prolactin, and 10% FBS. At the indicated time point during differentiation, cells were treated with 5 μg/ml LPS for 4 h.

### ShRNA/siRNA transfection

The shRNA plasmid (System Biosciences, USA) or siRNA was mixed with serum-free medium and then combined with the transfection reagent Lipofectamine 3000 (Thermo Fisher, USA). The resulting mixture was incubated with the cells for 12 h. After the silencing treatment, the medium was replaced with complete medium containing the appropriate pharmacological treatments, and the cells were further incubated for additional experiments. ShRNA/siRNA sequence and source are shown in Table S3.

### Cell Oil Red O staining

Differentiated HC11 cells were treated with 4% PFA for 30 min and then stained with filtered Oil Red O solution for 2 to 3 h at room temperature, protected from light. After excess stain was removed with 60% isopropanol, nuclei were stained with hematoxylin for 1 min, and lipid droplets were observed using an inverted microscope.

### Measurement of triacylglyceride concentration

Triglyceride content was measured using a kit (Nanjing Jiancheng, China). Differentiated HC11 cells were lysed in radioimmunoprecipitation assay buffer, and the lysates were collected by centrifugation. The optical density value at 510 nm was recorded, and triglyceride content was calculated based on the manufacturer’s instructions.

### Cell scratch assay

Undifferentiated HC11 cells were seeded in 6-well plates and cultured until reaching approximately 90% confluence. A scratch was created with a 200-μl pipette tip, and images were taken at the initial (0-h) time point. Following 24 h of treatment, the scratch area was reevaluated to analyze cell migration.

### Transwell assay

Undifferentiated HC11 cells, pretreated with the respective drugs, were seeded into the upper chamber of a transwell (Corning, USA) containing serum-free medium, while the lower chamber was filled with complete medium. After 24 h, migrated cells were stained with crystal violet and quantified.

### Real-time PCR

RNA was extracted from cells or tissues using RNA extraction kits (Ezbioscience, USA), and cDNA was synthesized using a reverse transcription kit. qPCR was performed using Color SYBR Green Master Mix (Ezbioscience, USA) and gene-specific primers. The thermal cycling conditions included an initial stage at 95 °C for 1 min, followed by 40 cycles of 95 °C for 10 s, 60 °C for 30 s, and 95 °C for 15 s. Melt curve analysis was performed after the PCR cycles. The primer sequences we used for real-time PCR are shown in Table S4.

### Western blotting

Proteins were separated by 10% sodium dodecyl sulfate–polyacrylamide gel electrophoresis and transferred onto polyvinylidene difluoride membranes. The membranes were blocked with nonfat milk and incubated with primary antibodies overnight at 4 °C. Following incubation with secondary antibodies, protein detection was performed using an enhanced chemiluminescence detection kit, and the band intensities were quantified using ImageJ software. The list of (immunofluorescence) antibodies used is provided in Table S1.

### Statistical analysis

Data were analyzed using one-way analysis of variance (ANOVA) with IBM SPSS 20.0. LSD (least significant difference) was applied for multiple comparisons. Results were presented as mean ± SEM (*n*). Statistical significance was indicated by *P < 0.05, **P < 0.01, and NS (*P* > 0.05). GraphPad Prism 9.0 and Adobe Illustrator (2024) were used for image analysis.

## Data Availability

All data generated or analyzed during this study are included in this published article (and its Supplementary Materials).
