# Supplementary material for: Omega-3 Fatty Acids Regulate Mammary Gland Lipogenesis and Development via Gαs-Mediated cAMP–EPAC Signaling Pathway
Source: Research (Wash D C). 2025 Jul 8;8:0767. doi: 10.34133/research.0767 (PMC12237497; doi:10.34133/research.0767)
Supplement: Supplementary 1 — Figs. S1 to S5 Tables. S1 to S4 [file research.0767.f1.zip › Supplementary Information.docx]

SUPPLEMENTARY MATERIALS

Figures S1 to S5


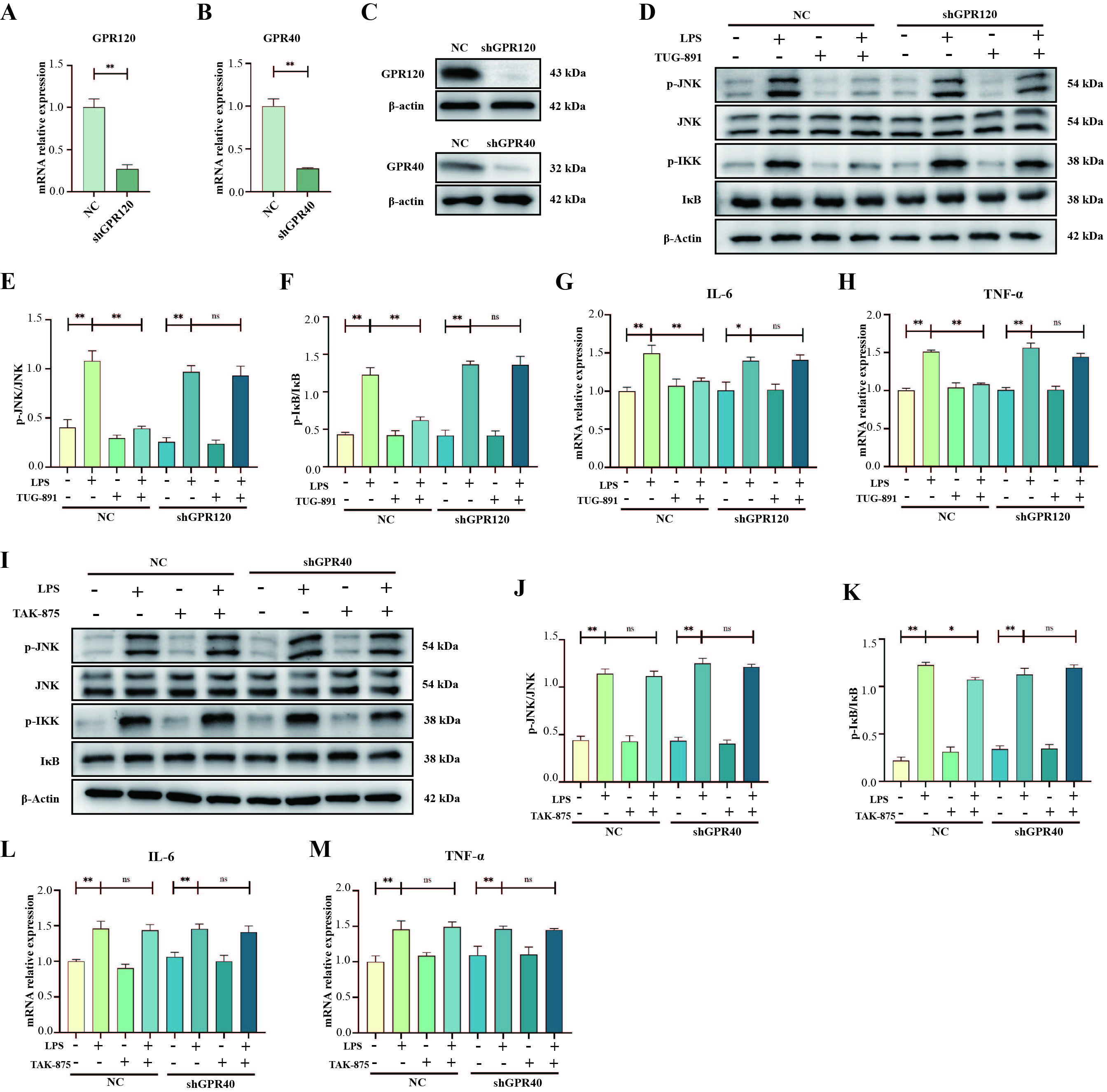


***Fig. S1.*** *Anti-Inflammatory Effects Mediated by GPR120 and GPR40 Agonists. (A-B) mRNA expression of GPR120 and GPR40 signaling proteins in HC11 cells with gene silencing, n = 3. (C) Western blot analysis of GPR120 and GPR40 signaling proteins in HC11 cells with gene silencing, n = 3.(D-F) Western blot analysis of inflammation-related signaling proteins (JNK/p-JNK and IκB/p-IKK) in HC11 cells with shRNA-GPR120 knockdown treated with TUG-891 (GPR120 agonist, 50 μM) and/or LPS (25 μg/ml), n = 3.(G-H) mRNA expression levels of inflammatory cytokines (IL-6 and TNF-α) in HC11 cells with shRNA-GPR120 knockdown treated with TUG-891 and/or LPS, n = 3. (I-K) Western blot analysis of inflammation-related signaling proteins (JNK/p-JNK and IκB/p-IKK) in HC11 cells with shRNA-GPR120 knockdown treated with TAK-875 (GPR40 agonist, 50 μM) and/or LPS, n = 3.(L-M) mRNA expression levels of inflammatory cytokines (IL-6 and TNF-α) in HC11 cells with shRNA-GPR120 knockdown treated with TAK-875 and/or LPS, n = 3. ^∗^ P < 0.05, ^∗∗^ P < 0.01. ns, not significant.*


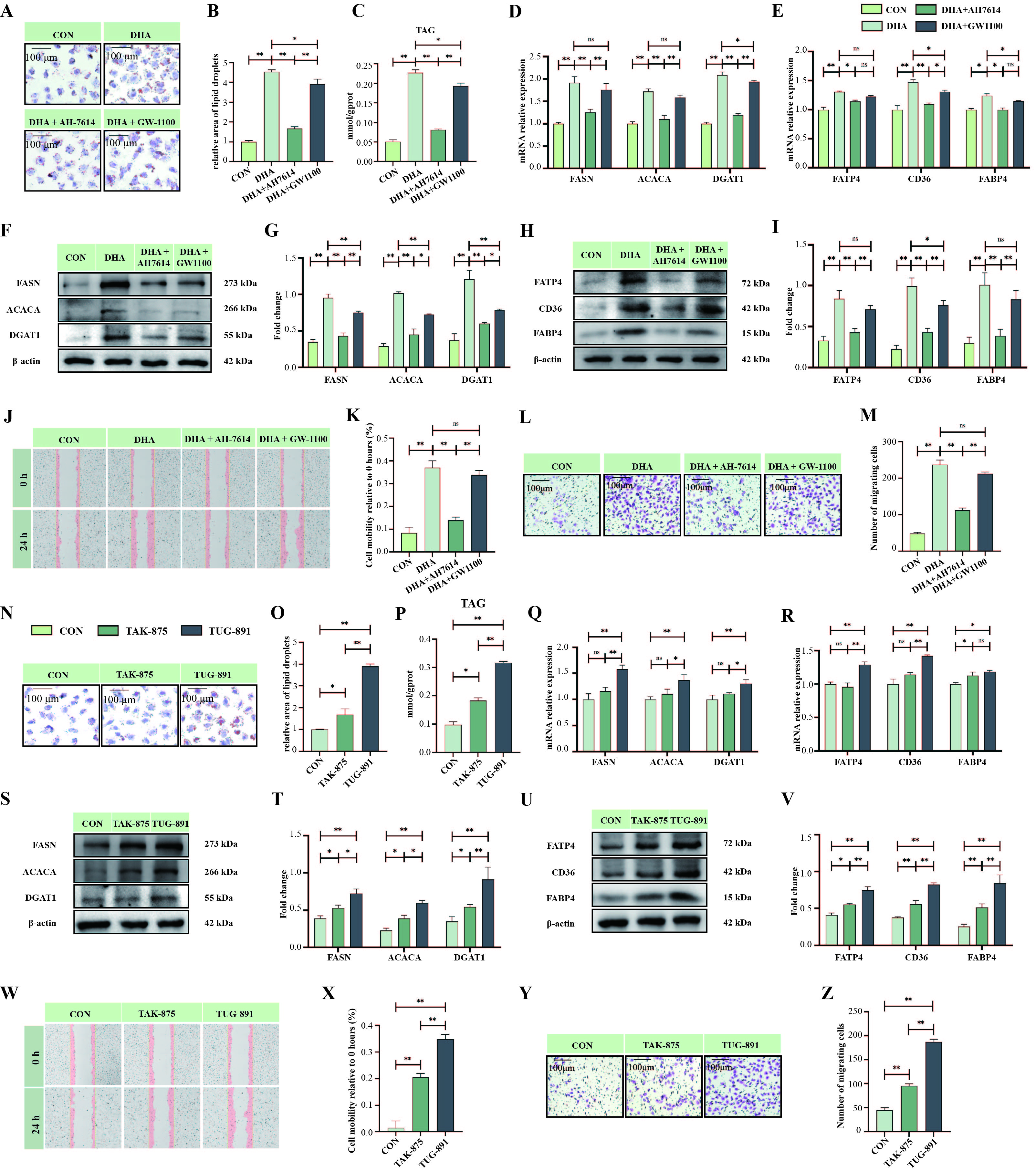


***Fig. S2****. Effects of GPR120 and GPR40 Inhibitors and Agonists on Milk Fat Synthesis and Cell Proliferation. (A–B) Oil Red O staining of HC11 cells treated with DHA (100 μM), AH-7614 (GPR120 inhibitor, 50 μM), and GW-1100 (GPR40 inhibitor, 50 μM), n = 3. Scale bar = 100 μm. (C) Triglyceride quantification in HC11 cells treated with DHA, AH-7614, and GW-1100, n = 3. (D-E) mRNA expression levels of lipid synthesis and transport proteins in HC11 cells treated with DHA, AH-7614, and GW-1100, n = 3. (F-I) Western blot analysis of lipid synthesis and transport proteins in HC11 cells treated with DHA, AH-7614, and GW-1100, n = 3. (J–K) Cell scratch assay results of HC11 cells treated with DHA, AH-7614, and GW-1100, n = 3. (L–M) Transwell assay of HC11 cells treated with DHA, AH-7614, and GW-1100, n = 3. Scale bar = 100 μm. (N–O) Oil Red O staining of HC11 cells treated with TAK-875 (GPR40 agonist, 50 μM) and TUG-891 (GPR120 agonist, 50 μM), n = 3. Scale bar = 100 μm. (P) Triglyceride quantification in HC11 cells treated with TAK-875 and TUG-891, n = 3. (Q-R) mRNA expression levels of lipid synthesis and transport proteins in HC11 cells treated with TAK-875 and TUG-891, n = 3. (S-V) Western blot analysis of lipid synthesis and transport proteins in HC11 cells treated with TAK-875 and TUG-891, n = 3. (W–X) Cell scratch assay results of HC11 cells treated with TAK-875 and TUG-891, n = 3. (Y–Z) Transwell assay of HC11 cells treated with TAK-875 and TUG-891, n = 3. Scale bar = 100 μm. ^∗^ P < 0.05, ^∗∗^ P < 0.01. ns, not significant.*


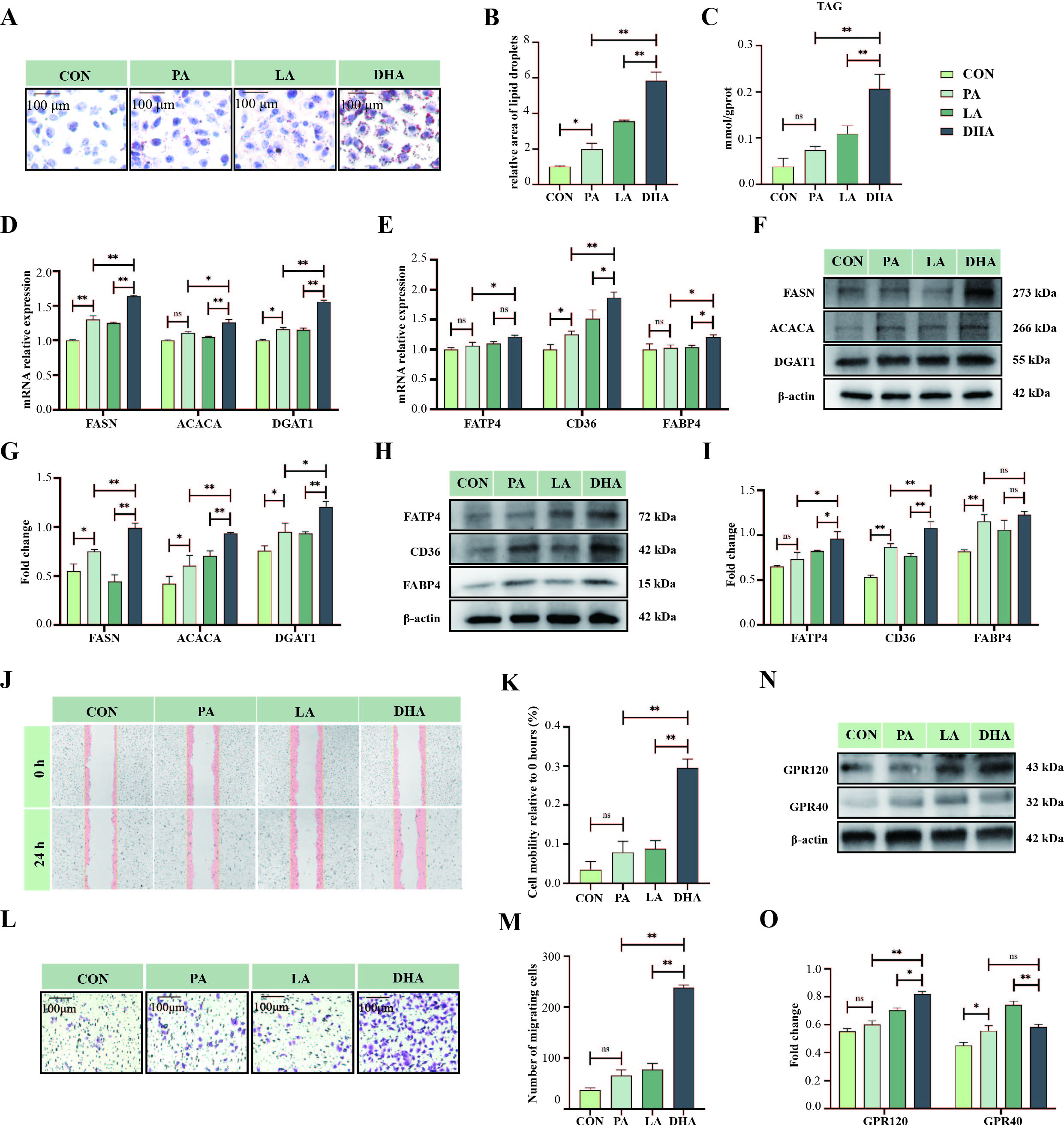


***Fig. S3.*** *Differential Effects of Saturated and Unsaturated Fatty Acids on Milk Fat Synthesis and Cell Proliferation. (A–B) Oil Red O staining of HC11 cells treated with PA (palmitic acid, 100 μM), LA (linoleic acid, 100 μM), and DHA (docosahexaenoic acid, 100 μM), n = 3. Scale bar = 100 μm.(C) Triglyceride quantification in HC11 cells treated with PA, LA, and DHA, n = 3. (D-E) mRNA expression levels of lipid synthesis and transport proteins in HC11 cells treated with PA, LA, and DHA, n = 3. (F-I) Western blot analysis of lipid synthesis and transport proteins in HC11 cells treated with PA, LA, and DHA, n = 3. (J–K) Cell scratch assay results of HC11 cells treated with PA, LA, and DHA, n = 3. (L–M) Transwell assay of HC11 cells treated with PA, LA, and DHA, n = 3. Scale bar = 100 μm. (N–O) Western blot analysis of GPR120 and GPR40 in HC11 cells treated with PA, LA, and DHA, n = 3. ^∗^ P < 0.05, ^∗∗^ P < 0.01. ns, not significant.*


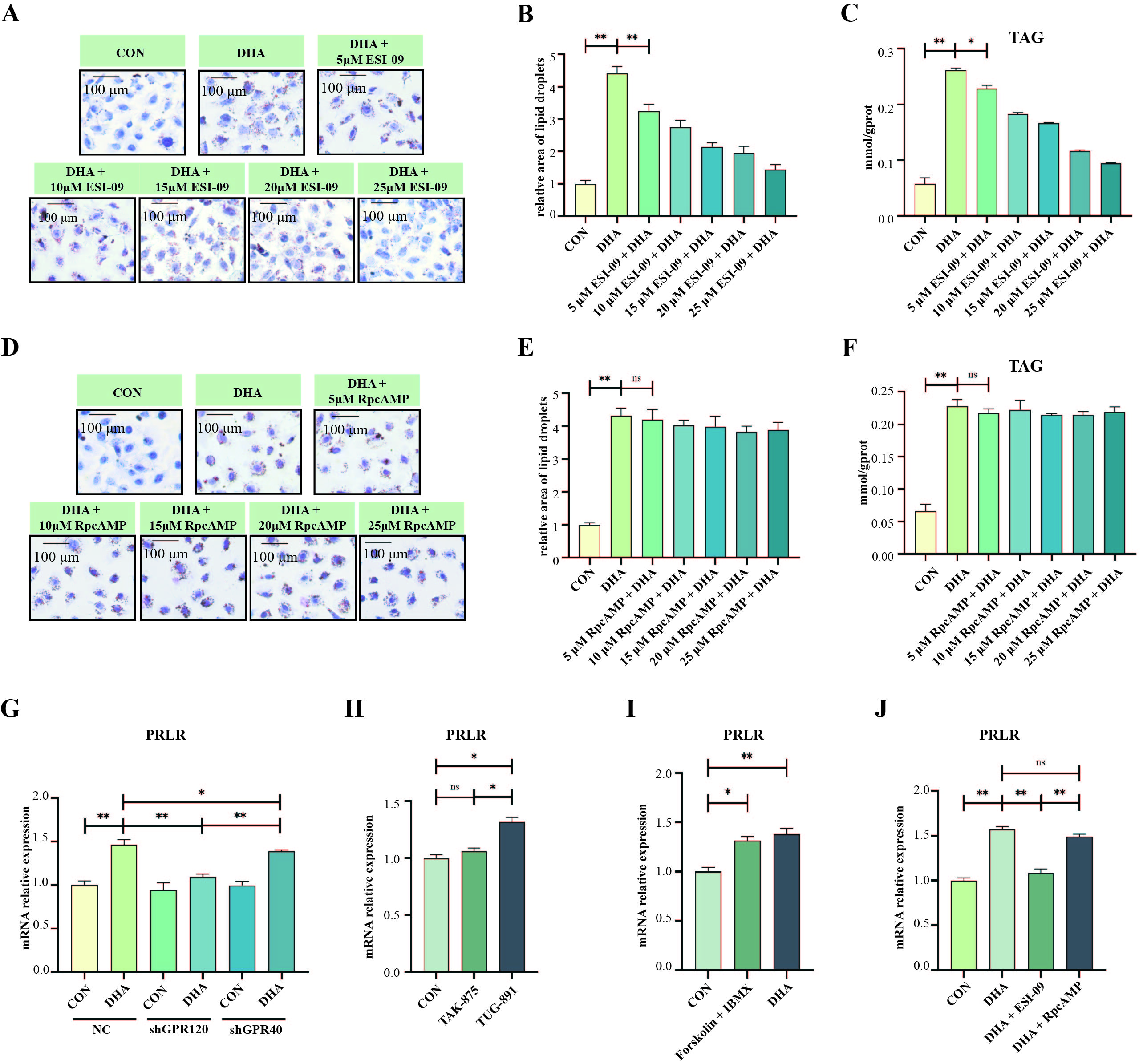
***Fig. S4.*** *Effects of EPAC and PKA Inhibition on Lipid Synthesis and PRLR Expression. (A–B) Oil Red O staining of HC11 cells treated with DHA and varying concentrations of ESI-09 (EPAC inhibitor), n = 3. Scale bar = 100 μm. (C) Triglyceride quantification in HC11 cells treated with DHA and varying concentrations of ESI-09, n = 3. (D–E) Oil Red O staining of HC11 cells treated with DHA and varying concentrations of RpcAMP (PKA inhibitor), n = 3. Scale bar = 100 μm. (F) Triglyceride quantification in HC11 cells treated with DHA and varying concentrations of RpcAMP, n = 3. (G–J) mRNA expression levels of PRLR in HC11 cells treated with DHA and inhibitors, n = 3. ^∗^ P < 0.05, ^∗∗^ P < 0.01. ns, not significant.*


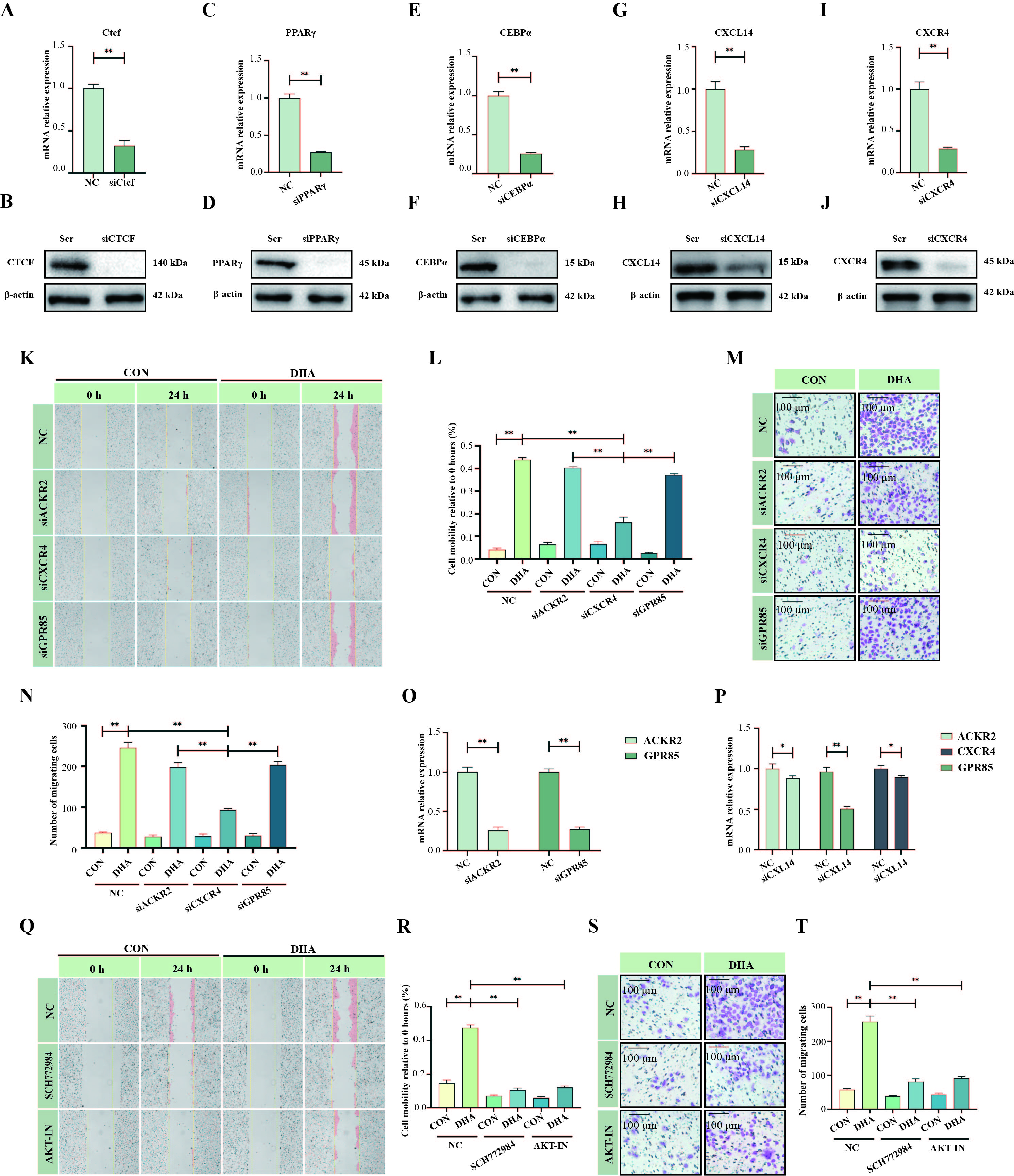
***Fig. S5.*** *Effects of ACKR2, CXCR4, and GPR85 Knockdown on Cell Proliferation. (A–J) Western blot analysis and mRNA expression of target genes in HC11 cells following siRNA knockdown, n = 3. (K–L) Cell scratch assay of HC11 cells treated with DHA (100 μM) under siRNA-ACKR2, siRNA-CXCR4, or siRNA-GPR85 knockdown conditions, n = 3. Scale bar = 100 μm. (M–N) Transwell migration assay of HC11 cells treated with DHA under siRNA-ACKR2, siRNA-CXCR4, or siRNA-GPR85 knockdown conditions, n = 3. Scale bar = 100 μm. (O) mRNA expression of ACKR2 and GPR85 following siRNA knockdown in HC11 cells, n = 3. (P) mRNA expression of ACKR2, CXCR4, and GPR85 in HC11 cells, n = 3. (Q–R) Cell scratch assay of HC11 cells treated with DHA in the presence of SCH772984 (ERK inhibitor) or AKT-IN (AKT inhibitor), n = 3. (S–T) Transwell migration assay of HC11 cells treated with DHA in the presence of SCH772984 or AKT-IN, n = 3. Scale bar = 100 μm. ^∗^ P < 0.05, ^∗∗^ P < 0.01. ns, not significant.*
