# Supplementary material for: Omega-3 Fatty Acids Regulate Mammary Gland Lipogenesis and Development via Gαs-Mediated cAMP–EPAC Signaling Pathway
Source: Research (Wash D C). 2025 Jul 8;8:0767. doi: 10.34133/research.0767 (PMC12237497; doi:10.34133/research.0767)
Supplement: Supplementary 1 — Figs. S1 to S5 Tables. S1 to S4 [file research.0767.f1.zip › Tables. S1 to S4.docx]

**SUPPLEMENTARY MATERIALS**

Tables 1 to 4

**Table 1 Antibody information**

| **Antibody** | **Company** | **Cat** | **Dilution ratio** |
| --- | --- | --- | --- |
| GPR120 (IF) | Santa Curz | sc-39752 | 250 |
| GPR40 (IF) | Abcam | ab236285 | 100 |
| Goat Anti-Rabbit IgG (IF) | Abcam | ab150083 | 500 |
| JNK | CST | 9252S | 1000 |
| p-JNK | CST | 4668S | 1000 |
| IκB | Abcam | ab32518 | 1000 |
| p-IKK | Abcam | ab133462 | 1000 |
| GPR120 | Santa Curz | sc-39752 | 1000 |
| GPR40 | Abcam | ab236285 | 1000 |
| FASN | Abcam | ab99539 | 1000 |
| ACACA | Abcam | ab72046 | 1000 |
| DGAT1 | Abcam | ab181180 | 1000 |
| FATP4 | Sangon Biotech | D123165 | 1000 |
| CD36 | Abcam | ab133625 | 1000 |
| FABP4 | Abcam | ab92501 | 1000 |
| CXCR4 | CST | 64837T | 1000 |
| CXCL14 | Abcam | ab264467 | 1000 |
| AKT | CST | 9272S | 1000 |
| p-AKT | CST | 9275S | 1000 |
| ERK | CST | 9102S | 1000 |
| p-ERK | CST | 9101S | 1000 |
| CTCF | CST | 2899S | 1000 |
| PPARγ | Abcam | ab178860 | 1000 |
| C/EBPα | CST | 2295S | 1000 |
| β-actin | Abcam | ab8226 | 2000 |
| Goat Anti-Rabbit IgG | Abcam | ab205718 | 5000 |
| Goat Anti-Mouse IgG | Abcam | ab205719 | 5000 |

**Table 2 Cell treatment reagent**

| **Reagent** | **Source** | **Identifier/ Cat#** |
| --- | --- | --- |
| DHA | Sigma | D2534 |
| PA | Sigma | P5585 |
| LA | Sigma | L1012 |
| LPS | Sigma | L2630 |
| TUG-891 | MCE | HY-100881 |
| TAK-875 | MCE | HY-10480 |
| AH-7614 | MCE | HY-19996 |
| GW-1100 | MCE | HY-50691 |
| Forskolin | MCE | HY-15371 |
| IBMX | MCE | HY-12318 |
| ESI-09 | MCE | HY-16704 |
| RpcAMP | MCE | HY-100530D |
| SCH772984 | MCE | HY-50846 |
| AKT-IN | MCE | HY-18296 |

**Table 3 sh/siRNA sequence**

| **sh/siRNA** | **Sense/ Anti-sense** | **Sequence** | **Source** |
| --- | --- | --- | --- |
| *GPR120* | Sense | GATCCCGAAATGACTTGTCTGTTATTCTCGAGAATAACAGACAAGTCATTTCGTTTTTG | System Biosciences |
|  | Anti-sense | AATTCAAAAACGAAATGACTTGTCTGTTATTCTCGAGAATAACAGACAAGTCATTTCGG |  |
| *GPR40* | Sense | GATCCCCTGGAGTGTGGTACTCAACTCGAGTTGAGTACCACACTCCAGGTTTTTG | System Biosciences |
|  | Anti-sense | AATTCAAAAACCTGGAGTGTGGTACTCAACTCGAGTTGAGTACCACACTCCAGGG |  |
| *CTCF* | Sense | GGAUGUCAACAGCAGUGUACA | RiboBio |
|  | Anti-sense | UACACUGCUGUUGACAUCCUG |  |
| *PPARγ* | Sense | AGGACUGUGUGACAGACAAGA | RiboBio |
|  | Anti-sense | UUGUCUGUCACACAGUCCUGU |  |
| *C/EBPα* | Sense | GAAGGAACUUGAAGCACAAUC | RiboBio |
|  | Anti-sense | UUGUGCUUCAAGUUCCUUCAG |  |
| *CXCL14* | Sense | GAGUGGUUCUGCAUAUUAAUG | RiboBio |
|  | Anti-sense | UUAAUAUGCAGAACCACUCGG |  |
| *CXCR4* | Sense | AGUUCUUAGUAGCUGUUUAUC | RiboBio |
|  | Anti-sense | UAAACAGCUACUAAGAACUUG |  |
| *ACKR2* | Sense | GCAGCUCCAUCUACGACUACG | RiboBio |
|  | Anti-sense | UAGUCGUAGAUGGAGCUGCUG |  |
| *GPR85* | Sense | GGCGAAGGUUUGUAAUUAAGA | RiboBio |
|  | Anti-sense | UUAAUUACAAACCUUCGCCAG |  |

**Table 4 Primer Sequences for Real-Time Quantitative PCR**

| **Gene** | **Direction** | **Sequence 5′-3′** | **Accession no.** |
| --- | --- | --- | --- |
| *GPR120* | Forward | ACCAAGTCAATCGCACCCAC | NM_181748.2 |
|  | Reverse | GTGAGACGACAAAGATGAGCC |  |
| *GPR40* | Forward | CATCACTCTGCCCCTGAAG | NM_194057.3 |
|  | Reverse | AAGGCAAAGACTGGGCAGA |  |
| *IL-6* | Forward | TACCACTTCACAAGTCGGAGGC | NM_001314054.1 |
|  | Reverse | CTGCAAGTGCATCATCGTTGTTC |  |
| *TNF-α* | Forward | GGTGCCTATGTCTCAGCCTCTT | NM_001278601.1 |
|  | Reverse | GCCATAGAACTGATGAGAGGGAG |  |
| *FASN* | Forward | AGCACTGCCTTCGGTTCAGTC | NM_007988.3 |
|  | Reverse | AAGAGCTGTGGAGGCCACTTG |  |
| *ACACA* | Forward | GAAGTCAGAGCCACGGCACA | XM_011248667.2 |
|  | Reverse | GGCAATCTCAGTTCAAGCCAGTC |  |
| *DGAT1* | Forward | CAGCTGTGGCCTTACTGGTTGA | NM_010046.4 |
|  | Reverse | CGGCACCACAGGTTGACATC |  |
| *FATP4* | Forward | GGTTACCTGTACTTCCGAGATC | NM_011989.5 |
|  | Reverse | CCTTTTTCAAGGTCTGTGCAAA |  |
| *CD36* | Forward | CGGCACCACAGGTTGACATC | NM_001159555.2 |
|  | Reverse | CGATCACAGCCCATTCTCCT |  |
| *FABP4* | Forward | CATCCGGTCAGAGAGTACTTTT | NM_001409513.1 |
|  | Reverse | TAGGGTTATGATGCTCTTCACC |  |
| *PRLR* | Forward | GCATCTTTCCACCAGTTCC | NM_001253781.1 |
|  | Reverse | AATTAGCCGCTCGTCCTCATT |  |
| *CXCR4* | Forward | CTTCCGGGATGAAAACGTC | NM_001356509.1 |
|  | Reverse | TGTCCGTCATGCTCCTTAGC |  |
| *CXCL14* | Forward | GAAGATGGTTATCGTCACCACC | NM_019568.2 |
|  | Reverse | CGTTCCAGGCATTGTACCACT |  |
| *CTCF* | Forward | AACCAGCCAACAGCCATCATTC | NM_001358924.2 |
|  | Reverse | GCCTGAGCCTCCTCTTCTTCC |  |
| *C/EBPα* | Forward | CAAGAAGTCGGTGGACAAGAACAG | NM_001287514.1 |
|  | Reverse | CGTTGCGTTGTTTGGCTTTATCTC |  |
| *PPARγ* | Forward | TGACTTGAACGACCAAGTAACTC | NM_001127330.3 |
|  | Reverse | CTAGTACAAGTCCTTGTAGATCTC |  |
| *ACKR2* | Forward | CTTCTTTTACTCCCGCATCG | NM_001276719.2 |
|  | Reverse | TATGGGAACCACAGCATGAA |  |
| *GPR85* | Forward | ATGCAGCCGACAACATTTTGC | NM_001330666.1 |
|  | Reverse | CAGGTGGAGCCATTTTTGACA |  |
| *β-actin* | Forward | CCACCATGTACCCAGGCATT | NM_007393.5 |
|  | Reverse | CGGACTCATCGTACTCCTGC |  |
